# The secreted protein PCYOX1L controls the surface expression of acid-sensing ion channel 1a

**DOI:** 10.1126/sciadv.adw4064

**Published:** 2025-07-25

**Authors:** Sven Kuspiel, Maria Schilling, Felicitas Weiß, Alison Wiesehahn, Lea Strüver, Günther Schmalzing, Dominik Wiemuth, Stefan Gründer

**Affiliations:** ^1^ Institute of Physiology, RWTH Aachen University, Aachen D-52074, Germany.; ^2^ Institute of Clinical Pharmacology, RWTH Aachen University, Aachen D-52074, Germany.

## Abstract

The modulation of neurotransmitter receptor density is an important molecular mechanism underlying synaptic plasticity. Acid-sensing ion channel 1a (ASIC1a), a receptor for protons, plays an important role in synaptic transmission. In this study, we used high-resolution proteomic analysis to identify prenylcysteine oxidase 1 like (PCYOX1L) as a hitherto unknown interaction partner of ASIC1a. We found that PCYOX1L is a secreted protein that promotes ASIC1a assembly. PCYOX1L was indispensable for ASIC1a expression in the plasma membrane and synaptosomes, and the genetic deletion of PCYOX1L severely impaired hippocampal long-term potentiation. Endocytosed PCYOX1L promoted plasma membrane expression of ASIC1a, and PCYOX1L secreted from astrocytes induced ASIC1a activity in neurons. Our findings reveal that a secreted protein, PCYOX1L, is a checkpoint for the plasma membrane expression of a synaptic ion channel.

## Teaser

A secreted protein controls the surface localization of a synaptic ion channel, influencing hippocampal long-term potentiation.

## INTRODUCTION

Synaptic plasticity is crucial for learning and memory, as it enables alteration of the strength of synaptic connections in response to activity. Acid-sensing ion channel 1a (ASIC1a) is a neuronal receptor for protons, which responds to pH changes in the synaptic cleft. However, compared to other neurotransmitter receptors, less is known about the molecular mechanisms that modulate ASIC1a density and function. ASICs are trimers; each subunit comprises two transmembrane domains (TMs) and a large extracellular domain (ECD) (*1*). The main ASICs in the brain are homomeric ASIC1a and heteromeric ASIC1a/2a (*2*), which operate at the proton concentrations reached in the synaptic cleft during synaptic transmission (*2*–*5*). ASIC1a is enriched in regions of the brain with strong excitatory input (*6*), localizes to the dendritic spines of hippocampal neurons (*7*, *8*), and contributes to excitatory postsynaptic currents at several glutamatergic synapses (*3*, *9*, *10*). Notably, ASIC1a plays an important role in synaptic plasticity, with genetic knockout (KO) of *Asic1* severely impairing hippocampal long-term potentiation (LTP) (*11*–*13*).

Unexpectedly, heterologously expressed ASIC1a mainly localizes to intracellular compartments, particularly the endoplasmic reticulum (ER) (*14*), indicating inefficient forward transport to the plasma membrane. In contrast, ASIC2a efficiently localizes at the plasma membrane (*14*, *15*) and targets ASIC1a to the postsynapse of hippocampal neurons (*16*). These findings suggest that different unknown mechanisms target homomeric ASIC1a to synapses, which may include auxiliary proteins forming complexes with ASIC1a.

To address this subject, here, we used high-resolution proteomic analysis to define the ASIC1a interactome, i.e., the set of proteins physically interacting with ASIC1a. We identified PCYOX1L as a secreted protein that tightly interacts with ASIC1a and promotes ASIC1a assembly and plasma membrane expression. Our results indicate that PCYOX1L acts as an unconventional chaperone for the postsynaptic ion channel ASIC1a, regulating its surface expression. Notably, PCYOX1L can perform this function transcellularly, establishing a hitherto unknown mechanism for receptor regulation.

## RESULTS

### PCYOX1L is a component of ASIC1a complexes

**Fig. 1.**
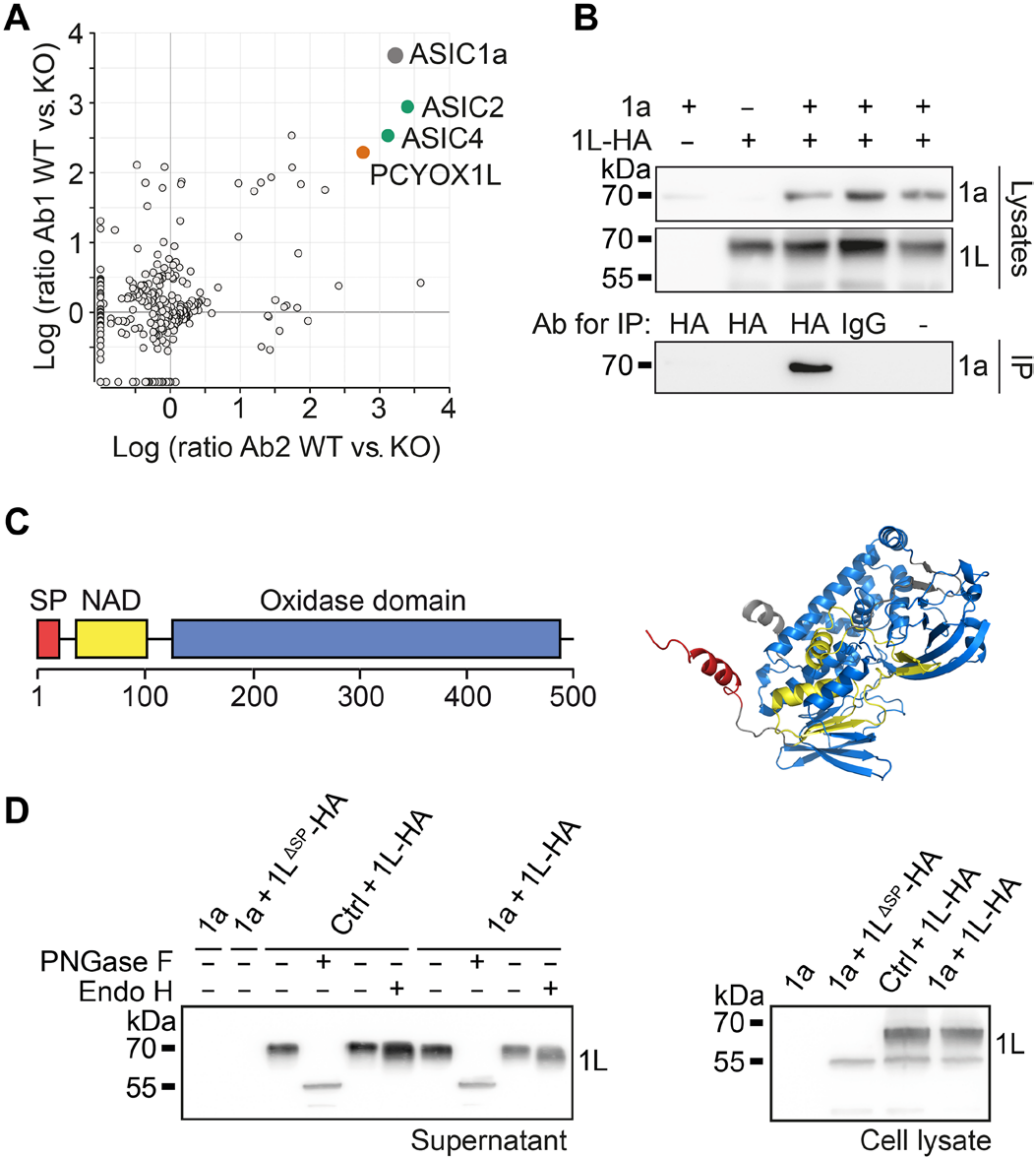
The secreted protein PCYOX1L is a component of ASIC1a complexes. (**A**) Log_10_ values of enrichment in *Asic*1^+/+^ versus *Asic*1^−/−^ brains of individual proteins co-purified with two ASIC1-specific antibodies. (**B**) Co-immunoprecipitation of ASIC1a with PCYOX1L-HA. Top: Expression controls. Bottom: Co-immunoprecipitation (co-IP) of ASIC1a with PCYOX1L-HA; co-IPs with rat IgG or no IgG (only beads) were performed as negative controls. (**C**) Domains and predicted structure of rat PCYOX1L. SP, signal peptide; NAD, FAD/NAD-binding domain. (**D**) Detection of PCYOX1L-HA in the supernatant of cells expressing the indicated proteins; the structurally unrelated α1 subunit of the glycine receptor, which assembles as functional homopentamer, was used as a control. Right: Western blot of cell lysates. 1a, rat ASIC1a; PNGase F, peptide *N*-glycosidase F; Endo H, endoglycosidase H; KO, knockout.

To characterize the ASIC1 interactome, we performed affinity purifications using two ASIC1-specific antibodies on solubilized membrane fractions prepared from whole brains of *Asic1*^+/+^ and *Asic1*^−/−^ mice. The purified ASIC1 complexes were analyzed by high-resolution liquid chromatography tandem mass spectrometry (LC-MS/MS), as previously described (*17*). In addition to the expected pore-forming subunits ASIC1a and ASIC2, ASIC1 complexes also contained ASIC4, suggesting that ASIC1a/4 heteromers constitute a hitherto underestimated ASIC fraction in the mouse brain. A protein of unknown function, prenylcysteine oxidase 1 like (PCYOX1L), was also consistently copurified in high abundance with both ASIC1-specific antibodies (Fig. 1A), suggesting that it is a robust constituent of ASIC1a complexes. Co-immunoprecipitation of proteins overexpressed in *Asic1*^−/−^ human embryonic kidney (HEK) cells confirmed the interaction between ASIC1a and PCYOX1L (Fig. 1B and fig. S1A).

Rat PCYOX1L comprises 494 amino acids, and sequence analysis suggests the presence of a proximal flavin adenine dinucleotide (FAD)/nicotinamide adenine dinucleotide (NAD)–binding domain and a large distal oxidase domain (Fig. 1C). AlphaFold (*18*) predicts that it folds into a compact globular protein comprising 16 α-helices and 18 β sheets (Fig. 1C and fig. S1B). Moreover, its N terminus exhibits a typical 20–amino acid ER signal peptide (SP) (Fig. 1C), suggesting that it is sorted into the ER and enters the secretory pathway. When PCYOX1L tagged with a hemagglutinin (HA) epitope was expressed in HEK cells, it was detected in the supernatant, and the deletion of the SP abolished PCYOX1L secretion (Fig. 1D). In addition, PCYOX1L contains five consensus sequences for N-glycosylation, and secreted PCYOX1L exhibited highly complex glycosylation (Fig. 1D). These results firmly established that PCYOX1L entered the secretory pathway via its SP and was released by HEK cells, suggesting that PCYOX1L could interact with the ECD of ASIC1a.

PCYOX1L is widely expressed in different tissues (fig. S1C) and was demonstrated to be important for bactericidal neutrophil activities (*19*). It is most closely related to prenylcysteine oxidase 1 (PCYOX1), with which it shares 44% sequence identity. PCYOX1 metabolizes prenylated proteins via its oxidase domain (*20*, *21*) and was shown to be secreted and to associate with lipoproteins, suggesting additional functions (*22*). Recently, it was reported that PCYOX1L also has prenylcysteine oxidase activity (*23*).

### PCYOX1L increases ASIC1a currents

**Fig. 2.**
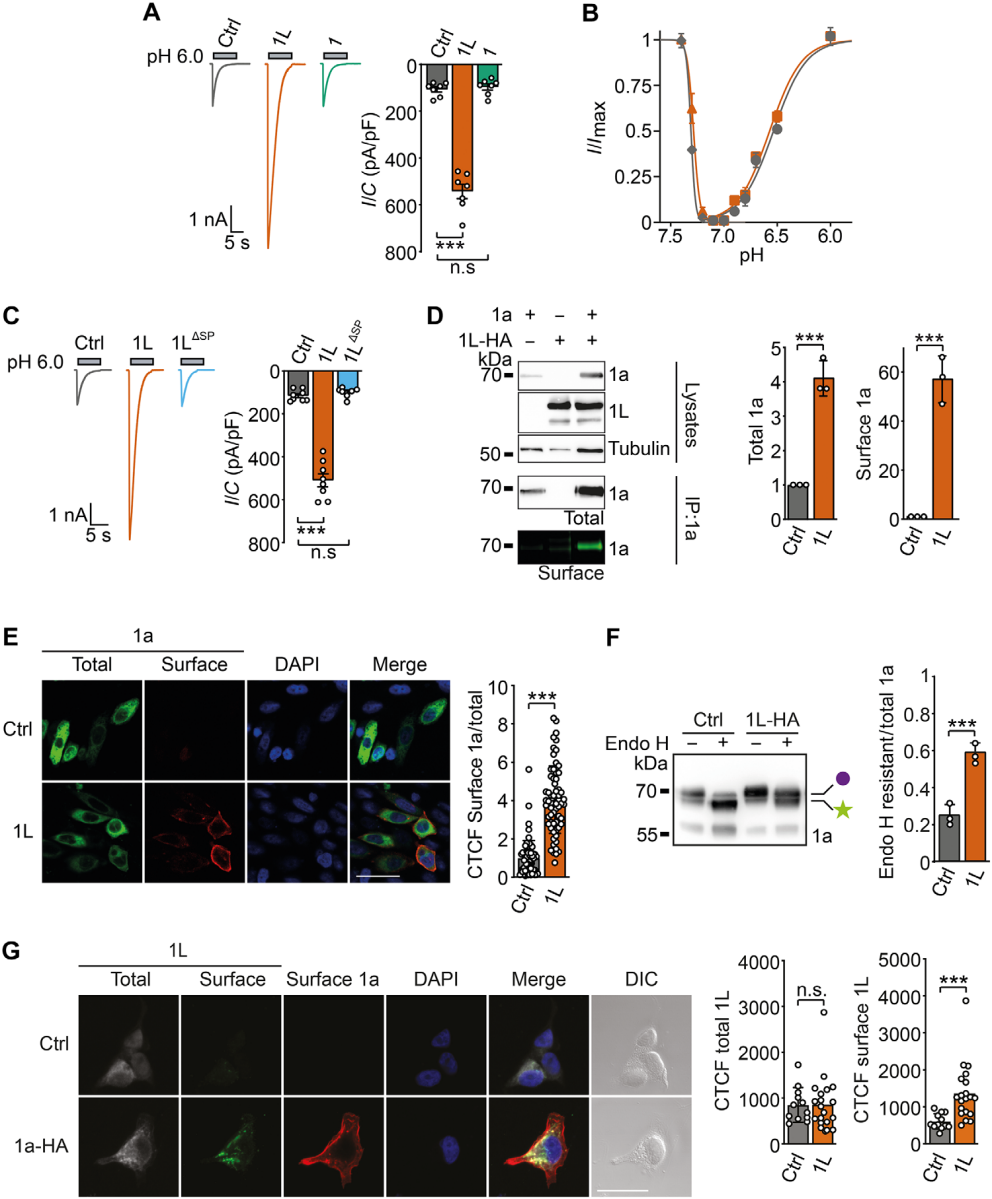
PCYOX1L potentiates ASIC1a currents by increasing its surface expression. (**A**) Representative traces (left) and quantification (right) of patch-clamp recordings of cells coexpressing ASIC1a with RFP (Ctrl), PCYOX1L, or PCYOX1; currents were elicited with pH 6.0 (*n* = 7). (**B**) Concentration-response curves of activation and steady-state desensitization of ASIC1a coexpressed with RFP or PCYOX1L (*n* = 5). (**C**) Representative traces (left) and quantification (right) of ASIC1a coexpressed with RFP (*n* = 7), PCYOX1L (n = 8), or PCYOX1L^ΔSP^ (*n* = 7). (**D**) Representative Western blots (left) and quantification (right) of ASIC1a in the absence and presence of PCYOX1L-HA. ASIC1a was immunoprecipitated and separated by PAGE, and the total ASIC1a pool detected with an anti-ASIC1 antibody and the surface pool with an infrared dye (*n* = 3). Western blots at the top show protein expression. (**E**) Confocal images of EGFP-ASIC1a-HA without (Ctrl) and with PCYOX1L. Scale bar, 10 μm. Right: Quantification of corrected total cell fluorescence (CTCF) of HA staining (surface ASIC1a) normalized to CTCF of EGFP staining (total ASIC1a). *N* = 3 independent experiments, each with *n* = 20 cells. (**F**) Glycosylation analysis of ASIC1a without and with PCYOX1L. Purple circle: endo H-resistant ASIC1a; green star: endo H-sensitive ASIC1a. Right: Quantification of endoglycosidase H–resistant ASIC1a to total ASIC1a (resistant plus sensitive) (*n* = 3). Endo H, endoglycosidase H. (**G**) Confocal images of cells expressing PCYOX1L-FLAG without and with EGFP-ASIC1a-HA. Scale bar, 10 μm. Right: Quantification of CTCF of total and surface PCYOX1L (*N* = 2 with *n* = 36 cells). Data were analyzed by ANOVA (A and C), unpaired *t* test (D and F), or Mann-Whitney *U* test (E and G). n.s., not significant; ***P < 0.001.

To assess whether PCYOX1L influences ASIC1a function, we conducted whole-cell patch-clamp recordings. To this end, we coexpressed rat ASIC1a with either red fluorescent protein (RFP) or rat PCYOX1L in HEK293 cells, in which endogenous ASIC1a was genetically knocked-out (*24*), and elicited currents with a saturating proton concentration (pH 6.0). PCYOX1L strongly potentiated typical transient ASIC currents (~5-fold; *P* < 0.001), suggesting an important effect on ASIC1a function (Fig. 2A). In contrast, the related protein PCYOX1 (which was not copurified with ASIC1a) did not increase ASIC1a currents (*P* = 0.54; Fig. 2A). PCYOX1L also potentiated currents of ASIC1b, but not ASIC2a or ASIC3 homomers (fig. S2A), demonstrating that PCYOX1L specifically interacted with ASIC1. PCYOX1L did not affect the biophysical properties of ASIC1a**—**including the pH_50_ of activation and steady-state desensitization (Fig. 2B), inhibition by amiloride, Ca^2+^ permeability, recovery from desensitization, tachyphylaxis, ion selectivity, and single-channel amplitude (fig. S2, B to G). ASIC1a current decay was slower in cells expressing PCYOX1L; however, this effect was nonspecific and due to the large current amplitudes in these cells (fig. S2, H and I). The overexpression of PCYOX1L also potentiated endogenous ASIC1a currents in HEK cells (fig. S3A). The deletion of the N-terminal SP abolished the effect of PCYOX1L on ASIC1a current amplitude (*P* = 0.14; Fig. 2C), confirming that it interacts with ASIC1a on the luminal/extracellular side. A recent study resolved the crystal structure of an ancestral PCYOX1 and identified two amino acids essential for its enzymatic function (*23*). The corresponding mutations in PCYOX1L (Y217A and M230W) did not impair its effect on ASIC1a (fig. S3B), indicating that the enzymatic activity of PCYOX1L is not required for its functional effect on ASIC1a.

The effect of PCYOX1L on ASIC1a current amplitude suggested that it might promote ASIC1a surface expression. To address this possibility, we coexpressed ASIC1a and PCYOX1L in HEK cells, labeled surface proteins with an infrared fluorescent dye, and immunoprecipitated ASIC1a. The presence of PCYOX1L strongly increased the surface pool of ASIC1a, but not of ASIC2a (Fig. 2D and fig. S4A); to a lesser extent, the total ASIC1a pool was also increased, suggesting that PCYOX1L increased the stability of ASIC1a (Fig. 2D). We confirmed increased surface expression using immunocytochemistry. We detected the surface pool of ASIC1a using an influenza HA epitope in the ECD and the total pool using a C-terminal fusion to green fluorescent protein (GFP) (ASIC1a^298HA^-GFP); the HA-tag between residues 298 and 299 does not change proton sensitivity nor subcellular distribution of ASIC1a (*8*) and did not affect the potentiation of ASIC1a currents by PCYOX1L (fig. S4B). ASIC1a surface expression was weak in the absence of overexpressed PCYOX1L and strongly increased when PCYOX1L was coexpressed (Fig. 2E). We assessed complex glycosylation as another measure of the surface-expressed ASIC1a pool and found an increased abundance of complex glycosylated ASIC1a in the presence of PCYOX1L (Fig. 2F), which is resistant to cleavage by endoglycosidase H (Endo H). In addition, we used a C-terminal FLAG epitope to detect the surface and total pools of PCYOX1L. Strikingly, PCYOX1L localized to the cell surface only when coexpressed with ASIC1a, suggesting that PCYOX1L interacts with the ECD of ASIC1a on the surface of living cells (Fig. 2G). Overall, four independent methods (current amplitudes, infrared staining combined with immunopurification, immunocytochemistry, and glycosylation pattern) consistently demonstrated that PCYOX1L increased ASIC1a surface expression in HEK293 cells.

### PCYOX1L controls ASIC1a surface expression

**Fig. 3.**
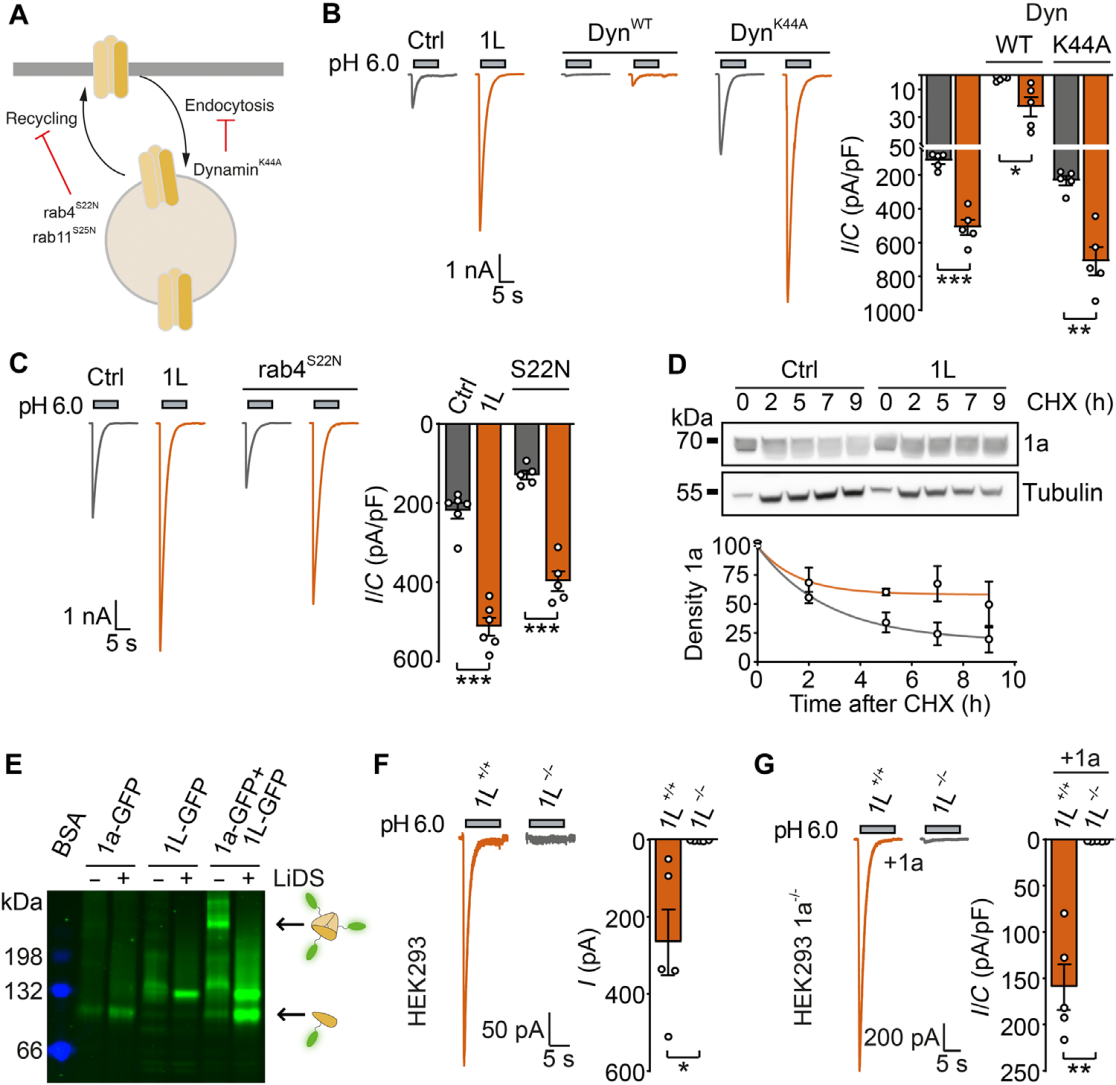
PCYOX1L has no effect on endocytosis or recycling but increases the abundance of ASIC1a trimers. (**A**) Cartoon illustrating the effect of dominant-negative mutants of proteins involved in endocytosis or recycling. (**B**) Representative traces (left) and quantification (right) of patch-clamp recordings of cells coexpressing ASIC1a with RFP or PCOX1L; cells additionally expressed dynamin or dynamin^K44A^ (*n* = 5). (**C**) As in (B), but with rab4^S22N^ instead of dynamin (*n* = 6 without rab4^S22N^; *n* = 5 with rab4^S22N^). (**D**) Top: WB analysis of cells coexpressing ASIC1a with ttcherry or PCYOX1L at different times after addition of 50 μM cycloheximide. Bottom: Densitometric analysis of total ASIC1a abundance normalized to tubulin (*n* = 3); abundance at time 0 was set as 100%. (**E**) High-resolution clear native-PAGE of ASIC1a-GFP, PCYOX1L-GFP, or both. ASIC1a trimers and monomers are indicated on the right. BSA (blue), which runs as a monomer, dimer, or trimer, served as size marker. Samples were also treated with lithium dodecylsulfate (LiDS). (**F**) Representative traces and quantification of endogenous ASIC currents from WT or Pcyox1l^−/−^ HEK cells (*n* = 5). (**G**) Representative traces and quantification of recordings from *Asic1a*^−/−^ or *Asic1a*^−/−^/*Pcyox1l*^−/−^ HEK cells expressing ASIC1a (*n* = 5). Data were analyzed by unpaired t test. *P < 0.05, **P < 0.01, and ***P < 0.001.

There are several possible mechanisms through which PCYOX1L may increase ASIC1a surface expression—including by stabilizing ASIC1a on the surface and inhibiting endocytosis, by enhancing ASIC1a recycling, and by enhancing ASIC1a expression and forward trafficking. We first tested for an effect on clathrin-mediated endocytosis, the primary pathway of ASIC1a internalization (*25*). To this end, we coexpressed ASIC1a with dynamin, which should promote endocytosis, or with a dominant negative mutant of dynamin (dynamin^K44A^), which should reduce endocytosis (Fig. 3A). As expected, ASIC1a current amplitudes were decreased by dynamin wild type (WT) and slightly increased by dynamin^K44A^. In both cases, PCYOX1L still potentiated ASIC1a currents (Fig. 3B), suggesting that PCYOX1L does not influence the clathrin-mediated endocytosis of ASIC1a. We next tested dominant negative mutants of rab4 and rab11, which are key regulators of fast and slow recycling, respectively (Fig. 3A) (*26*, *27*), which yielded similar results (Fig. 3C and fig. S5A), suggesting that PCYOX1L does not promote recycling of ASIC1a.

To examine the effects of PCYOX1L on ASIC1a stability, we performed cycloheximide (CHX) chase assays (*28*), which revealed increased ASIC1a stability in the presence of PCYOX1L (Fig. 3D). Last, we evaluated a possible role of PCYOX1L in ASIC1a oligomerization by performing high-resolution clear native PAGE (hrCN-PAGE) of GFP-tagged proteins. Unexpectedly, in the absence of PCYOX1L, ASIC1a existed almost exclusively as a monomer. In the presence of PCYOX1L, however, the portion of ASIC1a trimers strongly increased (Fig. 3E and fig. S5B). PCYOX1L increased the ratio of ASIC1a trimers to monomers, without increasing the pool of ASIC1a monomers, indicating increased trimerization of ASIC1a. In contrast, ASIC2a and ASIC3 trimers were readily detected in the absence of PCYOX1L, and their abundance was not increased by PCYOX1L (fig. S5, C and D). Moreover, the related protein PCYOX1 did not alter the abundance of ASIC1a trimers (fig. S5E). Together, these results suggest that PCYOX1L increases ASIC1a abundance and stability and promotes ASIC1a trimerization—leading us to conclude that PCYOX1L is involved in the assembly of ASIC1a.

These findings suggested that PCYOX1L may not only increase but also be necessary for ASIC1a surface expression. To test this, we used CRISPR-Cas9 to genetically knock out *Pcyox1l* in HEK cells (fig. S5, F and G). Strikingly, *Pcyox1l* KO completely abolished endogenous ASIC1a currents (Fig. 3F). Moreover, in HEK cells with a double KO of ASIC1a and PCYOX1L (*Asic1a*^−/−^/*Pcyox1l*^*−*/−^), the ASIC1a overexpression failed to rescue ASIC1a currents (Fig. 3G). Accordingly, *Pcyox1l*^*−*/−^ HEK cells exhibited no ASIC1a surface expression (fig. S5H), indicating that PCYOX1L is an auxiliary subunit of ASIC1a that is indispensable for ASIC1a surface localization in HEK cells.

### PCYOX1L is crucial for LTP

The Allen Brain Atlas indicates that PCYOX1L is expressed throughout the brain, at lower levels compared to ASIC1a expression (fig. S6A). In addition, the single-cell RNA sequencing database DropViz revealed the coexpression of PCYOX1L and ASIC1a in almost every neuronal cell type in the hippocampus, cerebellum, and cortex. Notably, although astrocytes do not express ASIC1a, they express PCYOX1L at levels similar to in neurons (fig. S6B).

**Fig. 4.**
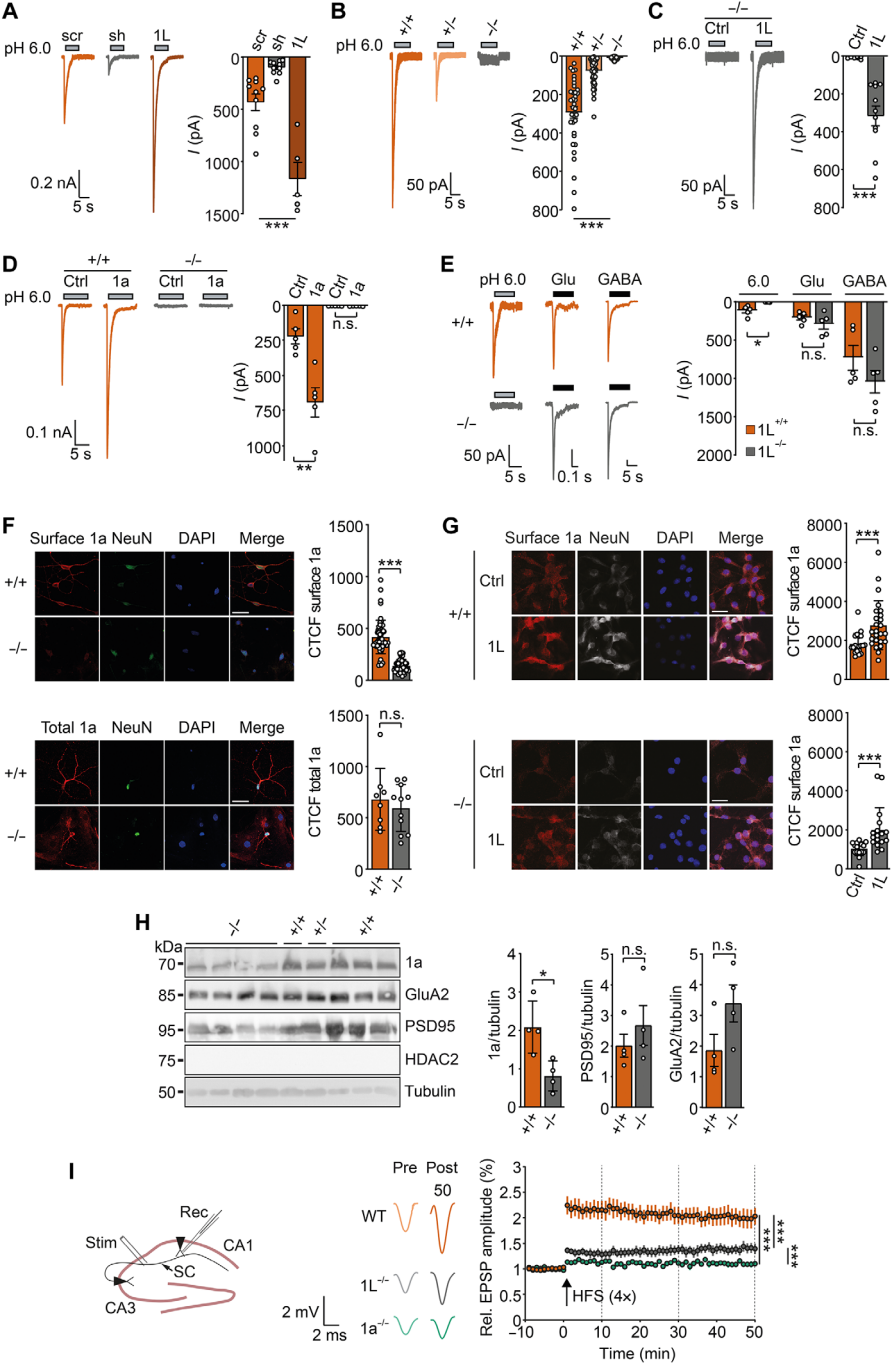
PCYOX1L is an indispensable auxiliary subunit of ASIC1a in neurons. (**A**) Representative traces (left) and quantification (right) of patch-clamp recordings of cortical neurons infected with scrambled RNA (*n* = 10) or shRNA against *Pcyox1l* (*n* = 10), or overexpressing mPCYOX1L (*n* = 5). *N* = 2 infections. (**B**) As in (A), but for cortical neurons from *Pcyox1l*^+/+^ (*n* = 35), Pcyox1l^+/−^ (*n* = 54), and Pcyox1l^−/−^ (*n* = 68) mice; *N* = 3 animals per genotype. (**C**) As in (A), but for cortical neurons from Pcyox1l^−/−^ mice infected with either mGL (Ctrl; *n* = 7) or mPCYOX1L (*n* = 11). *N* = 2 infections. (**D**) As in (A), but for neurons from Pcyox1l^+/+^ or Pcyox1l^−/−^ mice infected with mGL (Ctrl) or mASIC1a (*n* = 5). *N* = 2 animals per genotype. (**E**) As in (D), neurons were stimulated with pH 6.0, 1 mM glutamate, or 500 μM GABA (*n* = 5 neurons from two animals). (**F**) Left: Confocal images of cortical neurons from Pcyox1l^+/+^ and Pcyox1l^−/−^ mice stained for surface (top) or total ASIC1a (bottom). Only NeuN-positive cells showed intense red staining. Scale bar, 20 μm. Right: Quantification of CTCF of ASIC1a. Dots represent individual neurons (*n* = 121) from *N* = 2 independent cultures. (**G**) Left: Confocal images of CGCs of Pcyox1l^+/+^ and Pcyox1l^−/−^ mice overexpressing mPCYOX1L or mGL and stained for surface ASIC1a (red). Scale bar, 20 μm. Right: Quantification of surface ASIC1a intensity. Dots represent individual neurons (*n* = 78) from *N* = 2 independent cultures. (**H**) WB analysis of synaptosomal fractions of Pcyox1l^+/+^, Pcyox1l^+/−^, and Pcyox1l^+/+^ mice. *n* = 4 for +/+ and −/− mice; one mouse was heterozygous. The nuclear protein histone deacetylase 2 (HDAC2) was used as negative control. Right: Protein abundance in synaptosomes relative to tubulin. (**I**) Recordings of fEPSPs in whole-brain coronal slices from WT (*n* = 6), ASIC1^−/−^ (*n* = 6), and Pcyox1l^−/−^ (*n* = 5) mice; *N* = 4 animals per genotype. Data were analyzed by ANOVA (A, B, and I), unpaired *t* test (C to E and H) or Mann-Whitney *U* test (F and G).*P < 0.05, **P > 0.01, and ***P < 0.001.

We investigated the influence of PCYOX1L on ASIC1a currents in mouse cortical neurons and cerebellar granule cells (CGCs). First, we used adeno-associated viruses (AAVs) containing short hairpin RNA (shRNA) to knock down PCYOX1L in freshly isolated cortical neurons, which strongly reduced PCYOX1L expression (fig. S7A). At pH 6.0, cortical neurons infected with scrambled shRNA exhibited typical ASIC currents with an amplitude of 432.7 ± 76.2 pA (*n* = 10), while cortical neurons infected with PCYOX1L shRNA exhibited currents with markedly reduced amplitude (98.3 ± 19.4 pA; *n* = 10) (Fig. 4A). The PCYOX1L overexpression further increased the ASIC1 currents to 1167.0 ± 141.6 pA (*n* = 5; Fig. 4A), suggesting that PCYOX1L expression is a limiting factor determining ASIC current amplitude in cortical neurons.

To corroborate these findings, we used neurons from mice with genetic KO of *Pcyox1l*. Strikingly, pH 6.0 did not elicit any currents in cortical neurons from *Pcyox1l*^−/−^ mice (Fig. 4B). We observed a similar result in CGCs (fig. S7B). Cortical neurons from heterozygous *Pcyox1l*^+/−^ mice exhibited strongly reduced ASIC currents (*P* < 0.001; Fig. 4B), suggesting a dose-dependent effect of PCYOX1L on ASIC1a. The overexpression of mPCYOX1L in *Pcyox1l*^−/−^ neurons rescued ASIC currents (Fig. 4C), indicating that ASIC1a was present in these neurons but was not on their surface. Furthermore, while mASIC1a overexpression in *Pcyox1l*^+/+^ neurons increased ASIC current amplitude, it did not induce any ASIC currents in *Pcyox1l*^−/−^ neurons, emphasizing that ASIC1 surface expression in cortical neurons was dependent on the presence of PCYOX1L (Fig. 4D). These experiments did not affect the amplitudes of glutamate- and γ-aminobutyric acid (GABA)–evoked currents in cortical neurons from *Pcyox1l*^−/−^ mice (Fig. 4E). In the current clamp mode, application of pH 6.0 or 6.8 depolarized *Pcyox1l*^+/+^ neurons and elicited one or multiple action potentials, respectively, as previously reported (*29*). In contrast, acidic pH did neither depolarize nor generate action potentials in *Pcyox1l*^−/−^ neurons (fig. S7, C and D).

To detect endogenous ASIC1a on the surface of neurons, we used ASC06–immunoglobulin G (IgG), an antibody with high affinity for native membrane ASIC1a (fig. S7E) (*30*). While ASC06 stained the surface of cortical neurons from both *Pcyox1l*^+/+^ and *Pcyox1l*^−/−^ mice, the signal was strongly reduced for *Pcyox1l*^−/−^ mice (Fig. 4F). The staining of permeabilized neurons revealed comparable amounts of total ASIC1 in cortical neurons from *Pcyox1l*^+/+^ and *Pcyox1l*^−/−^ mice (Fig. 4F). This indicates that ASIC1a was present in neurons from *Pcyox1l*^−/−^ mice but was not transported to the plasma membrane, similar to our observations in HEK cells. The overexpression of mPCYOX1L in CGCs of *Pcyox1l*^+/+^ mice further increased ASIC1a staining (Fig. 4G). In CGCs from *Pcyox1l*^−/−^ mice, ASIC1a staining was low but increased upon mPCYOX1L expression (Fig. 4G). Overall, these results consistently demonstrate that PCYOX1L is an auxiliary protein of ASIC1a, which controls ASIC1a surface expression in HEK cells, cortical neurons, and CGCs.

To characterize the effect of PCYOX1L on ASIC1a abundance in synapses, we prepared synaptosomes from the brains of *Pcyox1l*^+/+^ and *Pcyox1l*^−/−^ mice. The ASIC1a abundance was strongly decreased in synaptosomes of *Pcyox1l*^−/−^ mice (Fig. 4H) but not in brain homogenates (fig. S7F), indicating that PCOX1L is crucial for the presence of ASIC1a in postsynaptic membranes. In contrast, the abundance of two other proteins of excitatory synapses, the AMPA-type glutamate receptor (AMPAR) subunit GluA2 and postsynaptic density protein 95 (PSD95), was not significantly changed in *Pcyox1l*^−/−^ mice, although there was a tendency for increased abundance (Fig. 4H).

*Asic1a*^−/−^ mice exhibit impaired LTP within the hippocampus (*11*–*13*). Upon finding that PCYOX1L was necessary for ASIC1a surface expression in neurons and synapses, we next examined whether *Pcyox1l* KO impaired hippocampal LTP. To this end, we stimulated Schaffer collaterals (SCs) and measured field excitatory postsynaptic potentials (fEPSPs) in CA1 neurons (Fig. 4I). In WT mice, high-frequency stimulation (HFS) potentiated fEPSP by 115%, and this potentiation slightly declined over time (Fig. 4I). In *Asic1a*^−/−^ mice, we observed only minor potentiation (11%; Fig. 4I), consistent with previous reports using the same HFS protocol (*12*, *13*). LTP was also strongly impaired in *Pcyox1l*^−/−^ mice (28% potentiation; *P* < 0.001), and this potentiation slightly increased over time to 40% (Fig. 4I). The difference in potentiation between *Asic1a*^−/−^ and *Pcyox1l*^−/−^ mice was significant (*P* < 0.001; Fig. 4I). These findings confirm that ASIC1a plays a role in synaptic plasticity and demonstrate that PCYOX1L is crucial for the role of ASIC1a in LTP.

### PCYOX1L can act transcellularly

**Fig. 5.**
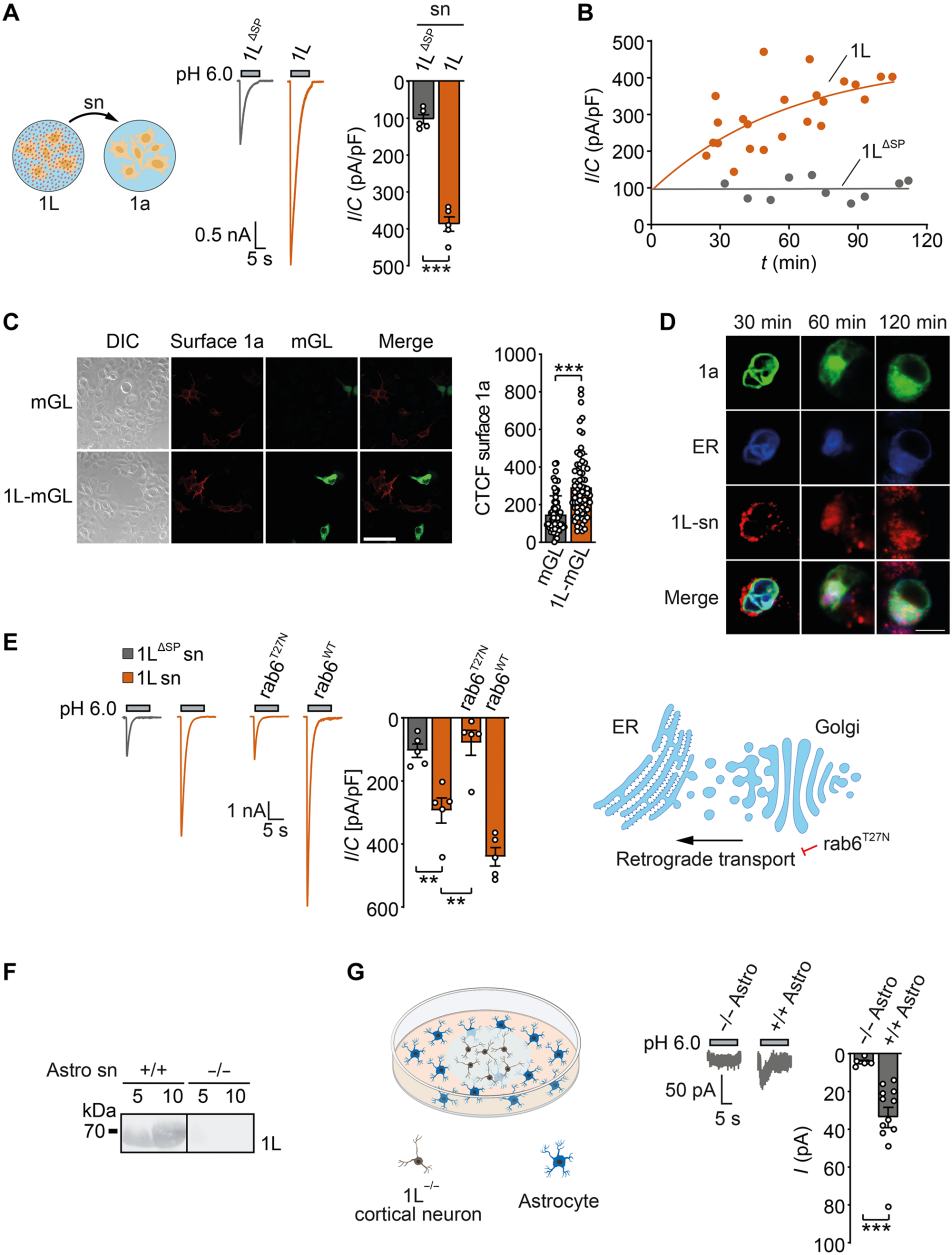
PCYOX1L can act transcellularly. (**A**) Representative traces (left) and quantification (right) of currents from ASIC1a-expressing HEK cells incubated for 120 min with the supernatants of cells expressing PCYOX1L or PCYOX1L^ΔSP^ (*n* = 5). (**B**) As in (A), but with incubation times between 30 and 120 min. A single-exponential function was fitted to calculate the time constant. (**C**) ASIC1a membrane staining (red) in ASIC1a-HA–expressing cells cocultured with cells expressing mGreen-Lantern (mGL) or PCYOX1L-mGL. Scale bar, 50 μm. Right, quantification of CTCF of membrane ASIC1a. *n* = 2 independent experiments with 60 cells each. (**D**) Confocal images of cells coexpressing ASIC1a-GFP (1a, green) and the ER marker-protein Sec61-BFP (ER, blue) and incubated with the supernatant from PCYOX1L-RFP–expressing cells (1L-sn, red) for 30, 60, or 120 min. Scale bar, 10 μm. (**E**) As in (A), but supernatant was additionally added to cells coexpressing ASIC1a with either rab6 or rab6^T27N^ (*n* = 5). Right: Cartoon illustrating retrograde transport by rab6 from the Golgi complex to the ER. (**F**) WB analysis of 5 or 10 μl of supernatant of astrocytes from *Pcyox1l*^+/+^ or *Pcyox1l*^−/−^ mice with an anti-PCYOX1L antibody. (**G**) Left: Cartoon illustrating coculture of neurons and astrocytes. Right: Representative traces and quantification of recordings from neurons cocultured with astrocytes from *Pcyox1l*^+/+^ (*n* = 5) or *Pcyox1l*^−/−^ mice (*n* = 11) from *N* = 2 animals of each genotype. Data were analyzed by unpaired *t* test (A and G), Mann-Whitney *U* test (C), or ANOVA (E). *P < 0.05, **P < 0.01, and ***P < 0.001.

Because PCYOX1L is secreted from HEK cells (Fig. 1D), we tested whether PCYOX1L could acutely potentiate ASIC1a currents when applied from the extracellular side. We applied the supernatant of PCYOX1L-expressing HEK cells to ASIC1a-expressing HEK cells during patch clamping and measured ASIC1a currents after 10 min. This protocol did not lead to an increased ASIC1a current amplitude (fig. S8A). On the other hand, when we applied supernatant from PCYOX1L-expressing cells to ASIC1a-expressing cells and measured currents after longer periods of time (up to 2 hours), ASIC1a currents were strongly potentiated (*P* < 0.001; Fig. 5, A and B). In contrast, the supernatant from cells expressing PCYOX1L^ΔSP^ did not increase the ASIC1a current amplitude. The observed increase in current amplitude was time dependent, with a time constant 𝜏 of ~60 min (Fig. 5B), suggesting that it was not mediated by a direct effect of PCYOX1L on ASIC1a present on the cell surface. In line with this interpretation, the coculture of ASIC1a-expressing HEK cells with PCYOX1L-expressing HEK cells led to strongly increased ASIC1a surface expression compared to the control (Fig. 5C). Moreover, applying the supernatant of PCYOX1L-expressing HEK cells to ASIC1a-expressing HEK cells resulted in strong intracellular PCYOX1L accumulation after 30 to 120 min (Fig. 5D). Over time, the fluorescent PCYOX1L signal increasingly overlapped with those of ASIC1a-GFP and the ER marker Sec61 (Fig. 5D and fig. S8B), revealing that endocytosed PCYOX1L reached the ER, where it could interact with ASIC1a.

Endocytic uptake and retrograde transport to the ER have previously been reported among AB-family toxins (e.g., Shiga and cholera toxins), which return to the ER via a rab6-mediated pathway (*31*, *32*). Rab6 regulates protein transport from the Golgi complex to the ER. We investigated the role of rab6 in the retrograde transport of PCYOX1L by applying the supernatant of PCYOX1L-expressing HEK cells to HEK cells expressing ASIC1a together with either rab6^WT^ or dominant-negative rab6^T27N^. Strikingly, the supernatant of PCYOX1L-expressing cells did not potentiate ASIC1a currents in rab6^T27N^-expressing cells but enhanced the currents of rab6^WT^-expressing cells to a greater extent than control cells (Fig. 5E). These findings suggested that endocytosed PCYOX1L indeed interacts with ASIC1a via a rab6-dependent pathway. Overall, our results confirmed the retrograde transport pathway of endocytosed PCYOX1L to the ER and strongly suggested that PCYOX1L increases ASIC1a surface expression through an interaction within the ER. In summary, these results show that PCYOX1L can be secreted from one cell and then internalized by another cell and reached the ER. Within the ER, PCYOX1L interacts with ASIC1a to promote its surface trafficking. Thus, PCYOX1L can modulate ASIC1a in a transcellular fashion.

Astrocytes express PCYOX1L but not ASIC1 (fig. S6B), and PCYOX1L was secreted from mouse astrocytes in culture (Fig. 5F). Therefore, we examined whether PCYOX1L secreted from astrocytes could enhance the surface expression of neuronal ASIC1a, by coculturing cortical astrocytes from either *Pcyox1l*^+/+^ or *Pcyox1l*^−/−^ mice with cortical neurons from *Pcyox1l*^−/−^ mice. A small but discernible transient ASIC current was elicited from neurons cocultured with *Pcyox1l*^+/+^ astrocytes, but not with *Pcyox1l*^−/−^ astrocytes (Fig. 5G). These results demonstrated that PCYOX1L from astrocytes can transcellularly regulate ASIC1 in neurons. To our knowledge, this is a hitherto unknown pathway for ion channel regulation in the central nervous system.

## DISCUSSION

The ion channel interactome**—**including pore-forming subunits and their auxiliary proteins**—**determines the density and function of synaptic ion channels (*33*, *34*). Although ASICs play important roles in synaptic transmission and LTP, their interactome has not yet been well defined (*35*). In the present study, we identified PCYOX1L as a protein tightly interacting with ASIC1a. PCYOX1L is secreted and functions in the ER to promote the assembly of homomeric ASIC1a, suggesting that it mainly functions as a chaperone. Strikingly, in the absence of PCYOX1L, plasma membrane expression of ASIC1a was abolished, indicating that PCYOX1L is indispensable for proper assembly of homomeric ASIC1a. PCYOX1L overexpression in HEK cells or neurons increased surface ASIC1a abundance and ASIC1a currents, while PCYOX1L reduction or KO reduced or abolished surface ASIC1a presence and currents, demonstrating that PCYOX1L functions as a checkpoint to control surface expression of ASIC1a homomers. The modulation of the surface expression of pore-forming subunits is a characteristic feature of auxiliary proteins; thus, the functions of PCYOX1L overlap with those of auxiliary proteins.

Some evidence suggested that PCYOX1L no longer interacts with ASIC1a after the assembly of homomeric ASIC1a. First, we did not detect any complex of ASIC1a trimers with PCYOX1L in native protein gels (Fig. 3E). Second, the functional properties of ASIC1a homomers did not differ in the presence versus absence of PCYOX1L (Fig. 2B and fig. S3, B to G). On the other hand, we detected more PCYOX1L at the cell surface in ASIC1a-expressing cells (Fig. 2G), suggesting an interaction between PCYOX1L and ASIC1a at the plasma membrane, and PCYOX1L increased the stability of ASIC1a (Fig. 3D). Thus, collectively, our data indicate that PCYOX1L mainly functions as a chaperone of ASIC1a, promoting the assembly of ASIC1a homomers, but has also characteristics of an auxiliary protein.

The assembly of other ligand-gated ion channels also requires chaperones. For example, in the first step of AMPAR assembly, monomers of the pore-forming GluA subunits tightly interact with the single-pass TM protein ABHD6 (*36*), such that ABHD6 overexpression prevents oligomerization (*37*). Interaction with the dimeric FRRS1l/CPT1c complex is required to release ABHD6 and promote GluR dimerization and tetramerization (*36*, *38*). In contrast, PCYOX1L overexpression enhanced the oligomerization of ASIC1a. Nicotinic acetylcholine receptor (nAChR) assembly is promoted by, or depends on (in the case of α7 nAChRs), the ER-resident four-pass TM protein NACHO (*39*, *40*). NACHO does not directly interact with nAChR subunits but rather associates with the ER oligosaccharyltransferase machinery and with the ER-resident single-pass TM chaperone calnexin, thereby promoting nAChR glycosylation (*34*, *41*). We do not yet know the precise mechanism by which PCYOX1L promotes ASIC1a assembly; however, it is unique and different from the known mechanisms of other chaperones of ligand-gated ion channels. In particular, other chaperones reside in the ER and typically have TM domains, indicating that PCYOX1L does not function only as a classical chaperone.

PCYOX1L is expressed in many tissues, suggesting that it may have functions unrelated to ASIC1a. The few studies that address the function of PCYOX1L have reported the following: Its deficiency reduces protein prenylation in (and viability of) mouse neutrophils, and it may be involved in autophagy and mitophagy (*19*); it is up-regulated in the blood of mice with deep vein thrombosis and aggravates thrombus formation (*42*), and it is a diagnostic biomarker for Alzheimer’s disease (*43*). Notably, none of these studies has clarified the precise functions of PCYOX1L. More is known about the related protein PCYOX1, which is involved in the catabolism of prenylated proteins (*20*, *21*, *44*). In addition, PCYOX1 has an N-terminal SP, is secreted from a hepatocyte cell line in association with lipoproteins (*45*), and accumulates in the arterial wall during atherogenesis (*22*), suggesting its further pro-oxidant activities. PCYOX1L exhibits conservation of the FAD/NAD-binding and lyase domains, suggesting that it might also have pro-oxidant activities. However, our results indicate that this enzymatic function is not required for promoting ASIC1a assembly (fig. S3B). There are other enzymes (e.g., ABHD6) that function as chaperones and auxiliary proteins of ion channels, independent of their enzymatic function (*36*, *38*).

Our experiments revealed that PCYOX1L KO abolished ASIC1a currents in neurons, reduced the presence of ASIC1a in synaptosomes, and severely impaired LTP, strongly suggesting that no ASIC1a homomers exist in postsynaptic membranes in the absence of PCYOX1L. Endocytosed PCYOX1L promoted ASIC1a plasma membrane expression in HEK cells and neurons, indicating that PCYOX1L can exert functions from one cell to another. A recent unbiased analysis of the synaptic proteome identified PCYOX1L as a novel synapse-enriched protein (*46*), confirming that PCYOX1L also localizes to synapses. Even more remarkably, we demonstrated that PCYOX1L secreted from astrocytes promoted ASIC1a plasma membrane expression in neurons lacking PCYOX1L (Fig. 5G), suggesting the exciting possibility that PCYOX1L-secreting astrocytes could influence synaptic transmission. The idea that astrocytes actively participate in synaptic activity and plasticity is at the core of the tripartite synapse concept (*47*), which expands the traditional view of synaptic communication to include a substantial role of glial cells in the brain.

Our study has certain limitations. Although PCYOX1L promoted the assembly and surface localization of ASIC1a, the precise molecular mechanism underlying this phenomenon remains unclear. Moreover, our study shows that, in principle, PCYOX1L could originate from different cells to control ASIC1a surface expression: from the same neuron or, after secretion, from a neighboring neuron or astrocyte. The degree to which the different sources contribute to the control of ASIC1a surface expression in vivo remains open. Future studies using mice with cell-specific KO will help to answer this question. Last, it is not clear whether and how the expression of PCYOX1L is regulated to dynamically control ASIC1a surface expression.

In summary, our results show that PCYOX1L controls ASIC1a oligomerization and thereby provides a checkpoint for neurons to adapt the pool of their plasma membrane-expressed ASIC1a homomers, revealing a previously unknown molecular mechanism for the regulation of a synaptic ion channel and of synaptic plasticity.

## MATERIALS AND METHODS

### Affinity purification MS

Solubilized membrane fractions from WT and ASIC1-KO mice were prepared as described (*48*, *49*). For preparative affinity purification, brain membranes were solubilized using ComplexioLyte 87 (CL-87; Logopharm, Freiburg, Germany; 0.8 ml/mg protein) supplemented with 2 mM EDTA/EGTA, iodoacetamide, phenylmethylsulfonyl fluoride, and protease inhibitors. After ultracentrifugation (S80 AT-3 rotor, 12 min at 45,000 rpm), cleared solubilisates were incubated with immobilized, CL-87 equilibrated antibodies (2 ml/15 μg; 2 hours at 4°C). After removal of unbound material, antibodies (Ab1: anti-ASIC1 OAAB05862 from AvivaSysBio, Ab2: anti-ASIC1 AGP-053 from Alomone) were washed twice for 5 min with CL-87 dilution buffer (Logopharm) and eluted with 2 × 7 μl of Lämmli buffer without reducing agents (10 min/37°C). A 1.7 μl of 1 M dithiothreitol was added to the eluates thereafter and incubated for 10 min at 37°C. Eluates were then loaded on an SDS–polyacrylamide gel electrophoresis (SDS-PAGE) gel and shortly run and silver-stained. Upper and lower molecular weight (MW) ranges were excised from each lane and subjected to tryptic digestion (*50*). LC-MS/MS analysis and identification of proteins were performed as described (*17*, *48*, *51*).

### Molecular biology

For coexpression of PCYOX1L and ASIC1a in HEK293 cells, we used rat ASIC1a. The DNA of rat PCYOX1L and rat PCYOX1 were synthesized by BioCat (Heidelberg, Germany). For site-directed mutagenesis, quick-change polymerase chain reaction (PCR) was performed using standard protocols. For electrophysiological analysis, we cloned PCYOX1L and ASIC1a into the pBudCE4.1 vector, which allows the independent expression of two genes from a single plasmid (*52*); PCYOX1L was under the control of the cytomegalovirus promoter and ASIC1a under the control of the human elongation factor-1α (EF-1α) promoter. All constructs are listed and described in Table 1.

**Table 1.** Plasmids used in this study.

Name	Species	Vector	Source or reference
ASIC1a	*Rattus norvergicus*	pcDNA3.1 (−)	S.G.’s laboratory
ASIC1a-GFP	*Rattus norvergicus*	pEGFP-N1	S.G.’s laboratory
EGFP-ASIC1a^298HA^	*Rattus norvergicus*	pEGFP-N1	Song *et al.* (*8*)
ASIC1a-GFP	*Homo sapiens*	pcDNA3.1 (−)	S.G.’s laboratory
ASIC1b	*Rattus norvergicus*	pcDNA3.1 (−)	S.G.’s laboratory
ASIC2a	*Rattus norvergicus*	pcDNA3.1 (−)	S.G.’s laboratory
ASIC2a-GFP	*Rattus norvergicus*	pEGFP-N1	S.G.’s laboratory
ASIC2a-FLAG	*Rattus norvergicus*	pEGFP-N1	S.G.’s laboratory
ASIC3	*Rattus norvergicus*	pcDNA3.1 (−)	S.G.’s laboratory
ASIC3-GFP	*Rattus norvergicus*	pEGFP-N1	S.G.’s laboratory
PCYOX1-GFP	*Rattus norvergicus*	pcDNA3.1 (−)	This study
PCYOX1L	*Rattus norvergicus*	pcDNA3.1 (−)	This study
PCYOX1L^ΔSP^	*Rattus norvergicus*	pcDNA3.1 (−)	This study
PCYXO1L-FLAG	*Rattus norvergicus*	pcDNA3.1 (−)	This study
PCYOX1L-HA	*Rattus norvergicus*	pcDNA3.1 (−)	This study
PCOX1L^ΔSP^-HA	*Rattus norvergicus*	pcDNA3.1 (−)	This study
PCYOX1L-GFP	*Rattus norvergicus*	pcDNA3.1 (−)	This study
PCYOX1L-RFP	*Rattus norvergicus*	pcDNA3.1 (−)	This study
PCYOX1L-mGL	*Rattus norvergicus*	pcDNA3.1 (−)	This study
PCYOX1L^Y217A^	*Rattus norvergicus*	pcDNA3.1 (−)	This study
PCYOX1L^M230W^	*Rattus norvergicus*	pcDNA3.1 (−)	This study
ASIC1a/RFP	*Rattus norvergicus*	pBudCE4.1	This study
ASIC1a/PCYOX1L	*Rattus norvergicus*	pBudCE4.1	This study
ASIC1a/PCYOX1L^ΔSP^	*Rattus norvergicus*	pBudCE4.1	This study
ASIC1a/PCYOX1	*Rattus norvergicus*	pBudCE4.1	This study
GlyR (α-subunit)	*H. sapiens*	pcDNA3.1 (−)	Sadtler *et al.* (*60*)
Dynamin	*H. sapiens*	pcDNA3.1 (−)	Damke *et al.* (*61*)
Dynamin^K44A^	*H. sapiens*	pcDNA3.1 (−)	Damke *et al.* (*61*)
Rab4^S22N^	*Drosophila melanogaster*	pcDNA3.1 (−)	Addgene plasmid #49476
Rab11^S25N^	*H. sapiens*	pcDNA3.1 (−)	Addgene plasmid #101046
Rab6	*H. sapiens*	pcDNA3.1 (−)	Addgene plasmid #46781
Rab6^T27N^	*H. sapiens*	pcDNA3.1 (−)	Addgene plasmid #46782
Sec61β	*H. sapiens*	pTagBFP-C1	Addgene plasmid #49154
mGreenLantern	*Aequorea victoria*	pcDNA3.1 (−)	Addgene plasmid #161912
AAV	–	pAAV-hSyn-MCS	van Loo *et al.* (*62*)

### Cell lines, cell culture, and transfections

HEK293 or HEK293 ASIC1a^−/−^ cells (provided by S. Pless) (*24*) were cultured in Dulbecco’s modified Eagle’s medium (DMEM) (PAN Biotech, Aidenbach, Germany) supplemented with 10% fetal bovine serum (FBS) (PAN Biotech) and 2.5 mM l-glutamine on 100-mm dishes and incubated at 37°C with 5% CO_2_. Cells were passaged every 3 to 4 days and before experiments.

To generate a PCYOX1L KO in HEK293 WT and HEK293 ASIC1a^−/−^ cells, the type II CRISPR-Cas9 system was used. Two overhanging strand breaks at a distance of 500 base pairs were induced by using two different Cas9 D10 nickases, as described (*53*). We induced the first double strand break at the end of intron 5 and the second at the beginning of exon 6, which contains most coding sequences. The targeted sequences were (excluding PAM-sequence): first target, 5′-GACCCCAGTAGGGAGTTCAG-3′, 5′-GGGTCTCGTTGTACCTCAAT-3′; second target, 5′-AGTGCAGACAGCTGAGTGGC-3′, 5′-GCTAAAGACCCTGTTCCGTT-3′, respectively. Single guide RNA pairs were cloned into the pX335A_hCas9(D10A) vector, which contains GFP. GFP-positive cells were sorted by fluorescence-activated cell sorting, and single cells were plated into 96-well plates. After expansion of cells, genomic DNA was isolated. The deletion of the genomic sequence was verified by PCR (5′-CACTTCCCGCTTTGCCCAGGAG-3′, 5′-ACCAAGTCTGAAGCACCTTGTGAAG-3′) and Sanger sequencing.

For electrophysiological recordings, we used HEK293 ASIC1a^−/−^ cells; only for the experiment reported in Fig. 3F and fig. S3A, we used HEK WT cells. Cells were seeded at a 1:10 to 1:20 dilution on glass cover slips in 35-mm dishes precoated with poly-d-lysine (0.1 mg/ml; Sigma-Aldrich, St. Louis, USA) for 10 min. After 1 hour, cells were transfected using calcium phosphate with 1 μg of DNA of each plasmid; for control experiments, we usually cotransfected RFP instead of PCYOX1L. Cells were recorded 1 day after transfection.

For immunoprecipitation, native hrCN-PAGE, and secretion experiments, cells were seeded on 35- or 100-mm dishes and transfected using calcium phosphate the following day. Medium was exchanged after 24 hours, and cells were harvested 24 hours later. For immuncytochemistry, cells were grown on poly-d-lysine–coated cover slips in 15.6-mm dishes. For CHX chase assays, cells were grown in 100-mm dishes. At 80% confluence, cells were transfected with constructs at a polyethylenimine (PEI)/DNA ratio of 3:1. Cells were used 24 hours after transfection.

For cocultivation of HEK cells, HEK 293 ASIC1a^−/−^ cells were transfected with ASIC1a-HA, mGreenLantern (mGL), or PCYOX1L-mGL. Twenty-four hours after transfection, cells were passaged, and cells transfected with ASIC1a-HA were grown together with cells transfected with either PCYOX1L-mGL or mGL (negative control) for an additional 24 hours.

### SDS-PAGE and Western blotting

Samples were incubated in 1× Laemmli sample buffer for 5 min at 95°C and separated by 10% SDS-PAGE under reducing conditions. Proteins were then transferred to a 0.4- μm pore size polyvinylidene difluoride membrane (Cytiva), which was blocked by 5% fat-free dry milk powder in tris-buffered saline (TBS) with 0.05% Tween (Sigma-Aldrich) (pH 7.5) (TBS-T). The membrane was incubated with primary antibodies in blocking solution overnight (o/n) at 4°C, washed in TBS-T, and probed with horseradish peroxidase (HRP)–conjugated secondary antibodies (1:12,000 in blocking solution) for 1 to 2 hours. After incubation and washing, protein bands were visualized using SuperSignal West Pico PLUS Chemiluminescent Substrate (Thermo Fisher Scientific, Waltham, USA) and detected using a chemiluminescence camera (Vilber, Eberhardzell, Germany). For CHX chase assay and assessment of endogenous PCYOX1L knockdown in cortical neurons, protein concentrations of the cell lysates were determined by bicinchoninic acid (BCA) assay. Equal amounts of protein (40 μg per sample) were used for Western blot (WB) analysis. All antibodies used in this study are listed and described in Table 2.

**Table 2.** Antibodies used in this study. WB, Western blot; co-IP, co-immunoprecipitation; IHC, immunohistochemistry.

Name	Source	Number	Clone	Dilution	Used in
Anti-ASIC1	BioLegend	MMS-5261	N271/44	1:1000	WB
Anti-ASIC1a	Quiang *et al.* (*30*)	–	ASC06	1:400	ICH
Anti-tubulin	Merck	T5161	B-5-1-2	1:1000	WB
Anti-PCYOX1L	Sigma-Aldrich	HPA037463	Polyclonal	1:1000	WB
Anti-HA	Roche	11867423001	3F10	1:1000	WB
				1:400	IHC
Anti-GluA2	Cell Signaling Technology	13607	E1L8U	1:1000	WB
Anti-HDAC2	Santa Cruz Biotechnology	Sc-81599	3F3	1:1000	WB
Anti-PSD95	Cell Signaling Technology	3450	D27E11	1:1000	WB
Anti-NeuN	Cell Signaling Technology	24307	D4G4O	1:1000	IHC
Anti-Flag	Cell Signaling Technology	14793	D6W5B	1:400	IHC
HRP goat anti-mouse IgG	Invitrogen	62–6520	Polyclonal	1:12500	WB
HRP goat anti-rabbit IgG	Invitrogen	31460	Polyclonal	1:12500	WB
HRP goat anti-rat IgG	Merck	AP136P	Polyclonal	1:12500	WB
Alexa Fluor 555 goat anti-rat IgG	Thermo Fisher Scientific	21434	Polyclonal	1:1000	IHC
Alexa Fluor 555 goat anti-human IgG	Thermo Fisher Scientific	A-21433	Polyclonal	1:1000	IHC
Alexa Fluor 488 goat anti-rabbit IgG	Thermo Fisher Scientific	A-11008	Polyclonal	1:1000	IHC

### Fluorescence labeling of ASIC surface pool

Forty-eight hours after transfection, HEK293 cells were washed with phosphate-buffered saline (PBS; pH 8.5). Surface proteins were labeled with the amine-reactive, membrane-impermeable fluorescent dye IRdye 800CW NHS-Ester (LI-COR, Lincoln, USA) in PBS (20 μg/ml, pH 8.5) for 60 min at 4°C. Cells were washed 4× with PBS (pH 8.5) and then lysed in 1% Triton X-100 in PBS. ASIC1a or ASIC2a-HA was precipitated and SDS-PAGE was performed. Wet gels were scanned using a fluorescence scanner (Cytiva, Marlborough, USA) to visualize the fluorescently labeled plasma membrane pool of ASICs. Next, Western blotting was performed, and protein quantities were determined by densitometric analysis (ImageJ).

### Co-immunoprecipitation/immunoprecipitation

Forty-eight hours after transfection, HEK293 cells were lysed with 1% Triton X-100 in PBS supplemented with complete protease inhibitor cocktail (Roche Diagnostics, Rotkreuz, Switzerland). Lysates were incubated with 1 μl of antibody for 1 hour at 4°C. A 25 μl of protein G-Sepharose beads (Sigma-Aldrich) was added and incubation continued for 1 hour at 4°C. Beads were collected by centrifugation and washed 4× in 1% Triton X-100 in PBS and analyzed by SDS-PAGE and Western blotting with the same antibody (immunoprecipitation) or antibodies against putative interaction partners (co-immunoprecipatation). Antibodies are listed in Table 2.

### Glycosylation analysis

For glycosylation analysis of secreted PCYOX1L, cell supernatants were incubated with antibody for 1.5 hours and then with 15 μl of G-Sepharose beads (Sigma-Aldrich) for 2 hours at 4°C. Beads were washed 6× with 1.2% Triton X-100 and resuspended. In parallel, ASIC1 of cell lysates was precipitated as described above. For each condition (supernatant and cell lysates), 18 μl was deglycosylated using the peptide *N*-glycosidase F and Endo H kit (New England Biolabs, Ipswich, USA); for control conditions, enzymes were replaced by ddH_2_O. After deglycosylation, beads were resuspended in 2.5× Laemmli sample buffer and subjected to SDS-PAGE and WB.

### hrCN-PAGE

hrCN-PAGE was performed as described (*54*, *55*). In brief, 48 hours after transfection, cells were lysed with homogenization buffer (0.1 M phosphate buffer, pH 8.0, 1% digitonin, 5 mM TCEP, 100 μM pefabloc, 50 μM antipain, 50 μM leupeptin, and 50 μM pepstatin) for 15 min on ice. Samples were centrifuged (13,000 rpm, 15 min at 4°C), and aliquots of the supernatant were resolved by hrCN-PAGE (4 to 16% polyacrylamide). Wet gels were scanned using a fluorescence scanner (Bio-Rad, Hercules, USA) to visualize the GFP-tags. Some samples were incubated for 1 hour at 37°C with 0.1% lithium dodecylsulfate for partial denaturation before being resolved by hrCN-PAGE.

### CHX chase assay

Before cell lysis, CHX (100 μM) was added, and cells were harvested at different time points (0, 2, 5, 7, and 9 hours). Cells were lysed at 4°C using RIPA buffer (10 mM Tris-HCl, pH 8.0, 1 mM EDTA, 0.5 mM EGTA, 1% Triton X-100, 0.1% Sodium Deoxycholate, 0.1% SDS, 140 mM NaCl) supplemented with protease inhibitors (Sigma-Aldrich). Lysates were centrifuged at 10,000*g* for 10 min at 4°C, and supernatants were subjected to SDS-PAGE and Western blotting.

### Whole-cell patch clamp

For electrophysiological recordings, bath solution (128 mM NaCl, 5.4 mM KCl, 2 mM CaCl_2_, and 1 mM MgCl_2_) with pH values between 7.4 and 6.0 was used. A 10 mM Hepes was used for pH > 6.6, and 100 mM MES was used for pH ≤ 6.6. The pipette solution (10 mM NaCl, 10 mM Hepes, 121 mM KCl, 5 mM EGTA, and 2 mM MgCl_2_) was adjusted to pH 7.25 and the osmolarity to 300 mosmol/kg using glucose. The osmolarity of the bath solution was 10 mosmol/kg higher than the pipette solution. Pipettes had a resistance of 3 to 5 milliohm. Solution exchange was gravity driven and controlled by the VC3-8xP series (ALA Scientific Instruments, Farmingdale, USA). Cells and neurons were clamped at −70 mV, and recordings were sampled at 10 kHz using an Axopatch 200B amplifier (Molecular Devices, San Jose, USA) and an analog-digital converter (Digidata 1440A, Molecular Devices) using Clampex 10.1 software (Molecular Devices).

For nonstationary noise analysis, cells were stimulated 30 to 80 times (10 s at pH 6.0 every 50 s). The first 10 to 15 recordings were excluded from analysis to rule out artifacts due to tachyphylaxis. Analysis was performed with “Ana” software (provided by Michael Pusch, Genova, Italy) as described (*56*). In brief, the variance of current was calculated and plotted against the current. Data were averaged into 40 sections and fitted with the following function

$$\sigma^{2}=\mathrm{iI}-\frac{I^{2}}{N}$$

with σ^2^ being the variance, *i* the apparent single channel amplitude, *I* the total current, and *N* the number of channels.

### Supernatant preparation

HEK293 cells were seeded on 100-mm dishes at a 1:10 dilution and transfected after 1 day with 3 μg of cDNA. Three days after transfection, the supernatant was harvested and concentrated 10- to 20-fold using Amicon Ultra Spin columns (Sigma-Aldrich). The supernatant of astrocytes was prepared likewise. For visualization of exogenous PCYXO1L-RFP and WB analysis of astrocyte supernatant, FluoroBrite DMEM (Thermo Fisher Scientific) without phenolred was used. For electrophysiological recordings, the medium of cells transfected with ASIC1a and GFP or rab6 was replaced with the supernatants, and cells were incubated for different time periods.

### Animal handling and PCYOX1L-KO mice

C57BL/6 N-*Pcyox1l*^em1(IMPC)J^/Mmucd mice were purchased from the Mutant Mouse Resource & Research Centers (MMRRC). C57BL/6 N-*Accn2* mice were a gift from J. A. Wemmie. Animal care was conducted under protocols approved by the State Office for Nature, Environment and Consumer Protection (LANUV) of North Rhine-Westphalia (NRW), Germany and was performed in accordance with LANUV NRW guidelines. Mice were housed in a conventional facility at 21°C on a 12-hour light/12-hour dark cycle with unrestricted access to food and water. Genotyping of C57BL/6 N-*Pcyox1l*^em1(IMPC)J^/Mmucd mice was conducted according to the MMRRC protocol for this strain. The following primers were used for genotyping PCR: 49020-mutF2 (5′-AACACTTCCCTCACTCTTGGCTAAGC-3′), 49020-comR2 (5′-CCTGCCACCCTCTTACTCGTCG-3′), and 49020-wtF (5′-ACAGTTCAATGCAGGCTCAG-3′). Genotyping of C57BL/6 N-*Accn2* mice was conducted according to standard PCR protocol using the following primers: ASIC53 (5′-CCGCCTTGAGCGGCAGGTTTAAAGG-3′), ASIC18, (5′-CATGTCACCAAGCTCGACGAGGTG-3′), and ASIC NEO (5′-TGGATGTGGAATGTGTGCGA-3′).

### Synaptosome isolation

Mice were anesthetized using an isoflurane vaporizer and quickly decapitated. Brains were removed and 10 ml of Syn-PER Synaptic Protein Extraction Reagent (Thermo Fisher Scientific) with 1% Phosphatase-Inhibitor cocktail (Sigma-Aldrich) and 1% Protease-Inhibitor cocktail (Sigma-Aldrich) per gram of brain were added. Brains were homogenized using a Dounce tissue grinder and centrifuged (1200*g*, 10 min, 4°C). A portion of the supernatant was saved for WB analysis (= homogenate). The rest of the supernatants was further centrifuged (15,000*g*, 20 min, 4°C), and the pellets were resuspended in 100 μl of Syn-PER reagent. Protein concentrations were determined using a BCA assay and adjusted to 6 μg/μl before WB analysis. Antibodies are listed in Table 2.

### ASC06 antibody production

The native ASIC1a-specific combinatorial antibody was produced as previously described (*30*). In brief, 20 100-mm dishes of HEK 293 cells were grown to 80 to 90% confluency and transfected with ASIC1-Ab6-GS HC Full IgG1 and ASIC1-Ab6-GS LC Lambda Full IgG1 using a PEI/DNA ratio of 4:1. Twenty-four hours after transfection, cells were transferred to agitation flasks and incubated at slow agitation in 100 ml of DMEM supplemented with 1.2% IgG-depleted FBS. Four days later, cells were pelleted (1000*g*, 5 min, room temperature), the pellet was discarded, and the supernatant was filtered using at 20-μm syringe filter. The supernatant was concentrated 20-fold with 10-kDa molecular weight cutoff (MWCO) concentration columns (Sartorius, Göttingen, Germany) and mixed 1:1 with binding buffer, containing 0.02 M sodium phosphate (pH 7). The human IgGs were purified using protein A affinity chromatography columns (Cytiva), according to the manufacturer’s instruction. The buffer was exchanged with PBS, and the antibody was desalted and concentrated with 10-kDa MWCO columns to a final volume of 500 μl. The protein concentration was determined using a BCA protein assay kit (Thermo Fisher Scientific). A 1% bovine serum albumin (BSA) and 0.01% sodium azide were added for long-term storage. The antibody was used at a concentration of 2 μg/ml.

### Immunohistochemistry in HEK cells and neurons

Neurons or HEK293 cells on glass coverslips were fixed with 4% paraformaldehyde plus 4% sucrose in PBS for 5 min at RT and washed 3× with PBS for 5 min. For surface staining, coverslips were incubated with 1× blocking solution (20% goat serum and 5% BSA in PBS) for 30 min at RT and then incubated with primary antibodies in 0.5× blocking solution at 4°C o/n. Cover slips were washed, followed by secondary antibody incubation (in 0.5× blocking solution) for 1 hour at RT. To stain all proteins, cells were permeabilized using 0.5% Triton in 1× blocking solution for 15 min at RT; antibody staining was performed as for surface staining. Nuclei were stained with 4′,6-diamidino-2-phenylindole at 1 μg/ml in PBS for 5 min at RT. Coverslips were then mounted on glass slides using mounting medium (Epredia Immu-Mount, Thermo Fisher Scientific).

Fluorescence imaging was performed using a LSM 700 confocal microscope (Zeiss). Digital images were prepared and analyzed using Fiji software (https://imagej.net/software/fiji/). Corrected total cell fluorescence (CTCF) was calculated as described (*57*). The CTCF signal of membrane staining was divided by CTCF signal of the GFP channel for normalization when using cells transfected with GFP-ASIC1a-HA fusion protein. For subcellular localization of exogenous PCYOX1L-RFP, images were prepared using the “ZEN” software (Zeiss) and Pearson correlation coefficients determined using the ImageJ Plugin “Coloco2”.

### AAV generation and production

Three AAV plasmids under the control of the human synapsin (hSyn) promoter were generated: mGreenlantern (pAAV-hSyn-mGL), mouse PCYOX1L (pAAV-mPCYOX1L), and ASIC1a with N-terminal tdTomato (pAAV-TOM-mASIC1a). In addition, AAVs to knockdown PCYOX1L (pAAV-hSyn-mGL-mU6-shRNA) and its corresponding scrambled version (pAAV-hSyn-mGL-mU6-scrambled) were designed using the BLOCK-iT RNAi Designer online tool (Invitrogen); the GC content was set to 30 to 55%. The sequence of the shRNA was 5′-gaattcGCAACAAGCTGGTTTGTTCttcaagagaGAACAAACCAGCTTGTTGCtttttgggatcct-3′, and the scrambled sequence was 5′-gaattcGTGTTCGTTCACGAACAGTttcaagagaACTGTTCGTGAACGAACACtttttgggatcct-3′ (sequences aligning to the target sequence are shown in upper case, and those forming a loop in lower case letters); sequences were flanked by restriction sites for Eco RI and Bam HI (underlined). The oligonucleotides were annealed and digested, and the shRNA and scrambled sequence were inserted into the plasmid. To visualize transfected neurons, mGreenLantern under the control of the mouse U6 promoter was cloned into the AAV vector using Cla I. Chimeric AAVs were produced as previously described (*58*). One week after isolation, neurons were infected with 10 μl of virus and incubated for at least 2 weeks before experiments.

### Culture of CGCs

Mice were euthanized on day P6 by decapitation, and the cerebella were extracted and placed in a petri dish containing ice-cold Hanks’ balanced salt solution (HBSS). The cerebella were cleaned from blood vessels and placed into a new petri dish containing ice-cold HBSS. Each cerebellum was cut in three pieces and washed using 1.5 ml of ice-cold HBSS. The HBSS was discarded, and 300 μl of trypsin/deoxyribonuclease (DNase) solution was added to the tube and incubated for 15 min at RT. The trypsin/DNase solution was discarded, the tissue pieces were washed 3× using 1.5 ml of ice-cold HBSS, and 300 μl of DNase solution was added. The cerebella were then triturated using three glass Pasteur pipettes with rounded tip, from wider to thinner diameter, until obtaining a single-cell suspension. The cells were centrifuged (15 min, 1000*g*, 4°C), and the cell pellet was resuspended in medium X-1 [neurobasal A containing 5% fetal horse serum (FHS), 1% penicillin/streptomycin, 0.1% BSA, insulin (10 μg/ml), 4 nM l-thyroxine, transferrin holo (100 μg/ml), 30 nM sodium-selenite, 1× B27 supplement, 2 mM l-glutamine, and 1 mM sodium pyruvate]. The cells were then diluted to a density of 1 to 2 × 10^5^ cells/ml for electrophysiology experiments or 1 to 2 × 10^6^ cells/ml for Western blot and seeded on PLL-coated six-well plates or glass coverslips and cultured for at least 24 hours.

### Culture of cortical neurons

Coverslips were coated with poly-l-ornithine (0.01 mg/ml in aqua dest., Sigma-Aldrich) for 30 min, washed with HBSS (Gibco, Carlsbad, USA), incubated with laminin (0.01 mg/ml in Dulbecco’s PBS, Sigma-Aldrich), and washed again. Coverslips were stored in HBSS o/n. Brains were isolated and stored in ice-cold HBSS. Meninges and blood vessels were removed carefully, and cortices isolated and incubated with 0.025% trypsin (Sigma-Aldrich) for 8 min (37°C). The tissue was washed with preparation medium (46.25 ml of neurobasal A, Thermo Fisher Scientific; 2.5 ml of horse serum, PAN Biotech; and 1.25 ml of l-glutamine, PAN Biotech; and penicillin/streptomycin, Sigma-Aldrich) and HBSS and kept in 600 μl of preparation medium. Tissue was triturated using the tip of 1-ml and 200-μl pipettes. A 100 μl was added to each cover slip (six per mouse). Cultures were stored at 37°C and 5% CO_2_ for 30 min. A 1 ml of culture medium was added (48.375 ml of neurobasal A; 1 ml of B27 supplement, Gibco; 0.5 ml of fetal calf serum, PAN Biotech; and 0.125 ml l-glutamine; penicillin/streptomycin). For maintenance of cells, culture medium was exchanged after 1, 2, and 5 to 7 days. From day 2, medium without antibiotics was used. A 800 μl of medium was replaced. After 5 to 7 days, neuronal cultures were further processed.

### Coculture of cortical neurons and astrocytes

First, astrocytes were isolated in a similar way as described for cortical neurons. The number of cortical neurons was reduced by adding a centrifugation step (1200*g* for 90 s) after each wash/trypsin step. In addition, the number of trituration steps was increased to three. Penicillin/streptomycin was used for 1 week instead of 1 day. This procedure yielded a dense glia cell culture with low amounts of cortical neurons. After 1 week, glial cells were separated as described (*59*). In brief, cultures were shaken at 180 rpm for 30 min, medium was replaced, and cultures were shaken again at 240 rpm for 6 hours. Medium was removed, and cells were treated with 0.25% trypsin for 5 min at 37°C and 5% CO_2_. Cells were collected and centrifuged at 180*g* for 5 min. Astrocytes were seeded into 24-well plates.

Cortical neurons from PCYOX1L^−/−^ mice were independently prepared and seeded on cover slips. They were then placed on top of astrocytes and cultured for 2 weeks before patch-clamp recordings.

### LTP recordings and slice preparation

Mice were anesthetized using an isoflurane vaporizer and quickly decapitated. Mice brains were removed quickly and equilibrated in ice-cold, calcium-free artificial cerebrospinal fluid (ACSF) (124 mM NaCl, 2 mM KCl, 1.25 mM KH_2_PO_4_, 2 mM MgSO_4_, 26 mM NaHCO_3_, and 10 mM dextrose) for 1 min. Coronal slices (400 μm) of whole brain were made using a vibratome (VT1000; Leica, Wetzlar, Germany). Brain slices were incubated in calcium-containing (2 mM) ACSF perfused with carbogen at 27°C. The temperature was raised by 1°C every 5 min to 32°C, and slices were incubated for 1 hour and then transferred to an interface perfusion chamber (custom-made, gift of H. Luhmann, Mainz). Slices were equilibrated in the chamber for 1 hour before electrophysiological recordings. Stimulation electrode (bipolar wolfram electrode, self-made) was placed at Schaffer collaterals from CA3. Recording electrode (glass pipette filled with ACSF, resistance ~1 milliohm) was placed into postsynaptic endings at CA1 (see Fig. 4I). Synaptic strength curve was determined, and baseline was recorded at 30% of maximal fEPSP amplitude for 10 min. EPSPs were recorded with a frequency of 0.066 Hz. Four stimulations within 1 min were averaged. HFS was applied by stimulating 4× with 100 Hz for 1 s every 20 s, and EPSPs were recorded for 50 min. The EPSP amplitude was normalized to the mean EPSP amplitude of baseline. Slices were recorded from four different animals per genotype.

### Visualization of data from dropviz

Transcript levels of ASIC1a, ASIC2a, and PCYOX1L were downloaded from the DropViz website, and log values were converted into transcripts per 100,000 transcripts. Transcript levels of subcluster for the same cell type were averaged and visualized using GraphPad Prism.

### Statistical analysis

We assumed normal distribution of electrophysiological data and densitometric analysis of western blots but did not formally check for it. The sample sizes (*n*) indicate the number of individual cells (HEK293 cells or neurons). Cells were randomly allocated to the experimental groups. For experiments using HEK293 cells, we used cells from at least two independent transfections. For experiments using confocal microscopy or neurons, we indicate the number *N* of independent experiments or individual animals in the figure legends. Bar graphs in figures show the means ± SEM, but individual data points are shown, allowing to assess the variation of the data. *P* values were determined by a two-sided Student’s *t* test or, for comparison of CTCF values, by a Mann-Whitney *U* test or, for comparison of >2 groups, by an analysis of variance (ANOVA), followed by Tukey’s test. Recordings of isolated cortical neurons from PCYOX1L KO mice (Fig. 4B) and LTP recordings (Fig. 4I) were performed blinded.
