# Supplementary material for: The secreted protein PCYOX1L controls the surface expression of acid-sensing ion channel 1a
Source: Sci Adv. 2025 Jul 25;11(30):eadw4064. doi: 10.1126/sciadv.adw4064 (PMC13155533; doi:10.1126/sciadv.adw4064)
Supplement: Supplementary file 1 — Figs. S1 to S8 [file sciadv.adw4064_sm.pdf]

Science Advances
AAAS

Supplementary Materials for

The secreted protein PCYOX1L controls the surface expression of acid-sensing ion channel 1a

Sven Kuspiel *et al.*

Corresponding author: Dominik Wiemuth, dwiemuth@ukaachen.de; Stefan Gründer, sgruender@ukaachen.de

Sci. Adv. **11**, eadw4064 (2025)
DOI: 10.1126/sciadv.adw4064

This PDF file includes:

Figs. S1 to S8

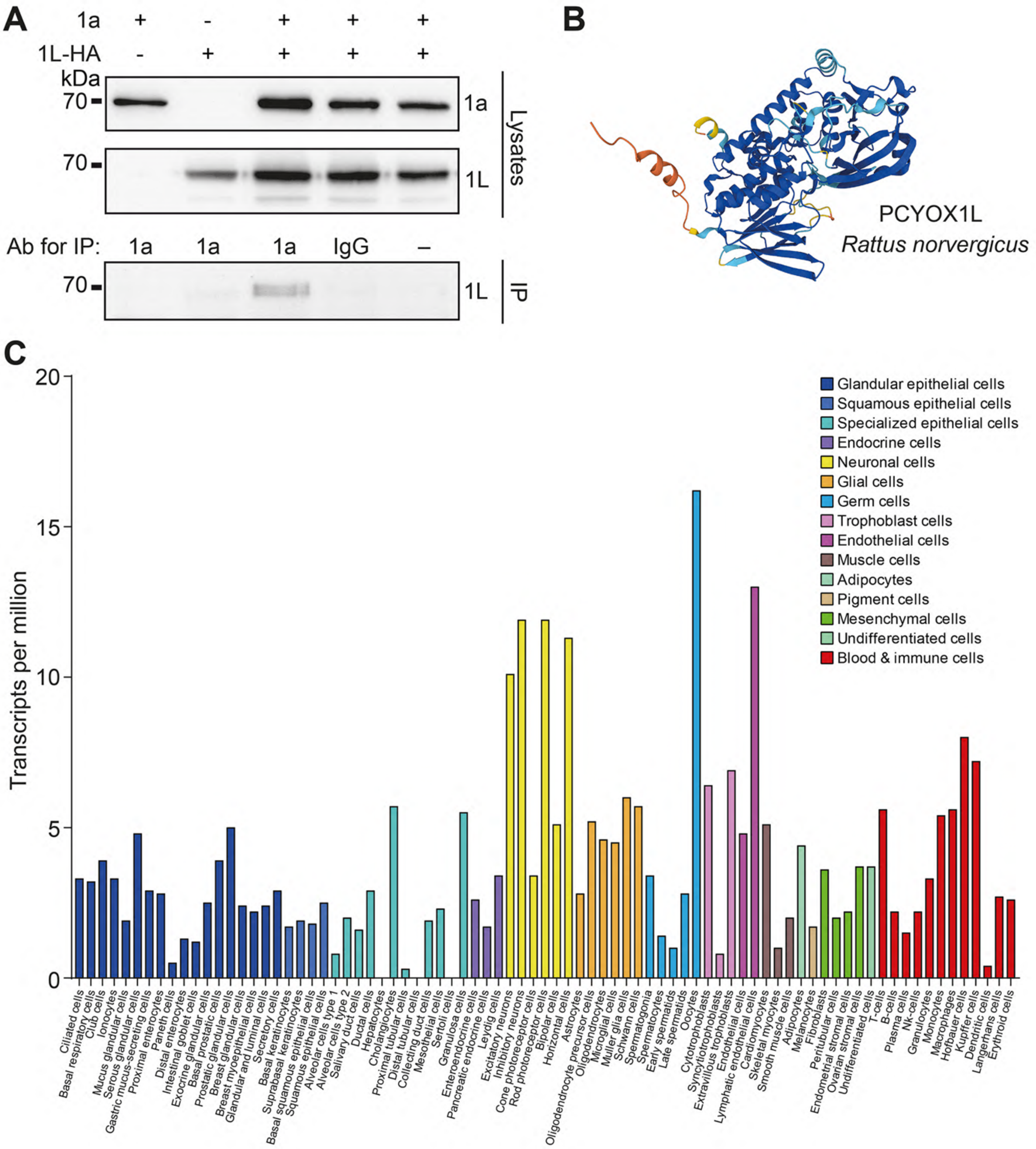

Fig. S1. PCYOX1L is predicted to be a globular protein and is ubiquitously expressed in the human body. (**A**) Co-immunoprecipitation of PCYOX1L-HA with ASIC1a. Top, expression controls. Bottom, co-IP; co-IPs with mouse IgG or no IgG (only beads) were performed as negative controls. (**B**) AlphaFold structure of rat PCYOX1L with indicated confidence score (plDDT): > 90, dark blue; 70-90, blue; 50-70, yellow; < 50, orange. The orange α-helix with a low score represents the signal peptide. (**C**) Expression of PCYOX1L in single cell-types of the human body. Raw data was obtained from the Human Protein Atlas (www.proteinatlas.org).

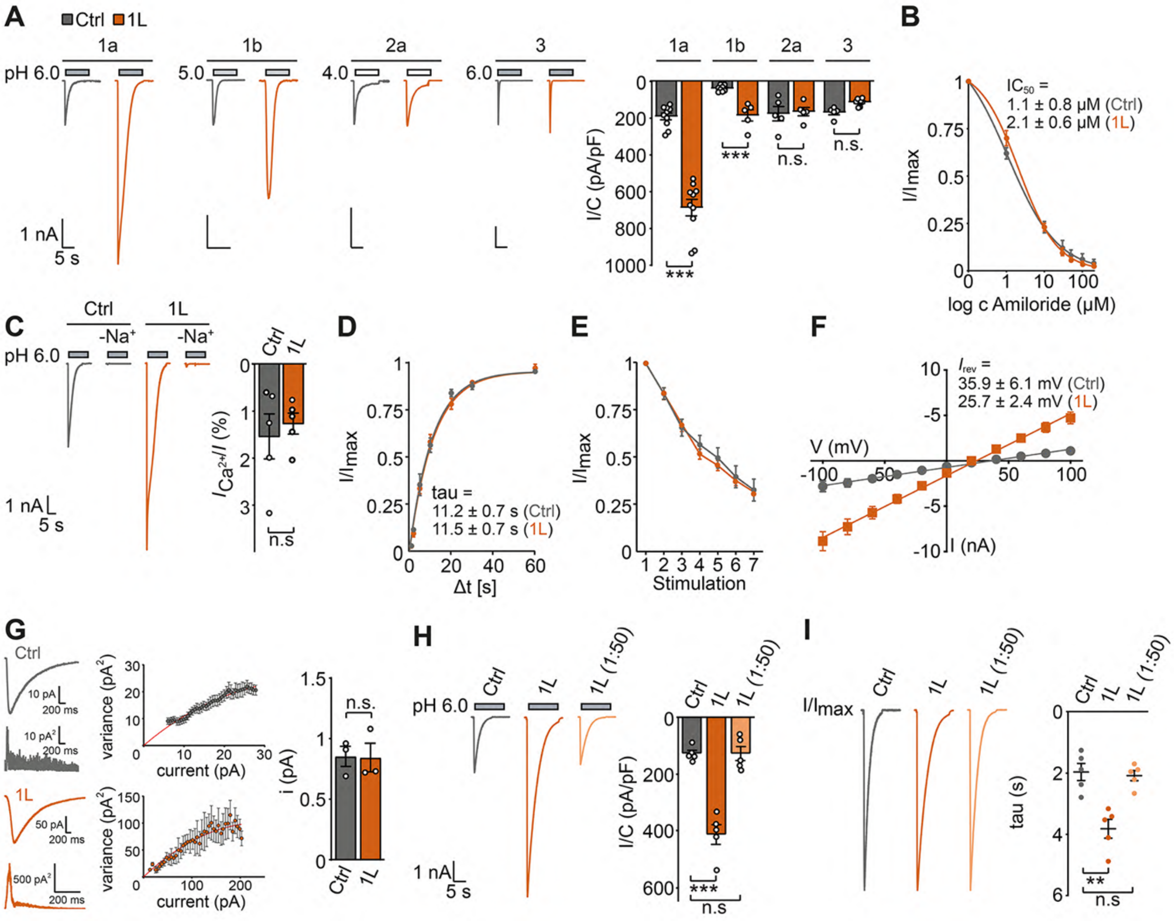

Fig. S2. PCYOX1L does not alter the biophysical properties of ASIC1a. (**A**) Representative traces (left) and quantification (right) of patch-clamp recordings of cells co-expressing ASIC1a, ASIC1b, ASIC2a, or ASIC3 together with RFP or PCYOX1L. Stimulation using pH 6.0, 5.0, or 4.0, as indicated (number of recorded cells from left to right: 10, 10, 8, 5, 5, 5, 5, 6). (**B to I**) ASIC1a was co-expressed with RFP or PCYOX1L and stimulated with pH 6.0. (**B**) Inhibition by increasing concentrations of amiloride (1 – 100 µM; n = 5). (**C**) Current amplitudes with and without Na^+ in the extracellular solution; Na^+ was substituted with N-Methyl D-Glucamine (NMDG) (n = 5). (**D**) Time between two stimulations varied between 1 and 60 s to assess recovery from desensitization; currents were normalized to the current after 60 s recovery (n = 5). (**E**) Cells were stimulated seven times consecutively to assess tachyphylaxis. Each stimulation was normalized to the first stimulation (n = 5). (**F**) voltage ramp protocol (from -100 mV to 100 mV in 1 s) was applied at approximately 90% of peak amplitude (n = 5). (**G**) Left, representative mean current and mean variance traces of cells co-expressing ASIC1a with RFP or PCYOX1L. Middle, mean variance plots. Right, single channel amplitude at -70 mV (n =3). (**H**) Cells transfected with diluted ASIC1a/PCYOX1L DNA (1:50) showed the same current amplitudes as cells expressing undiluted ASIC1a without PCYOX1L. Cells transfected with diluted ASIC1a (1:50) did not respond to low pH, probably due a lack of ASIC1a surface expression (data not shown; n = 5). (**I**) Analysis of the desensitization of the measurements displayed in (H). Left, current traces scaled to the amplitude of ASIC1a plus PCYOX1L. Right, time constants of desensitization. High ASIC1a current amplitudes in the presence of PCYOX1L result in an apparent slowing of ASIC1a desensitization. Bars represent the mean ± S.E.M. Data were analyzed by unpaired *t* test (A, C and G) or ANOVA (H and I). n.s., not significant; **, $P < 0.01$; ***, $P < 0.001$.

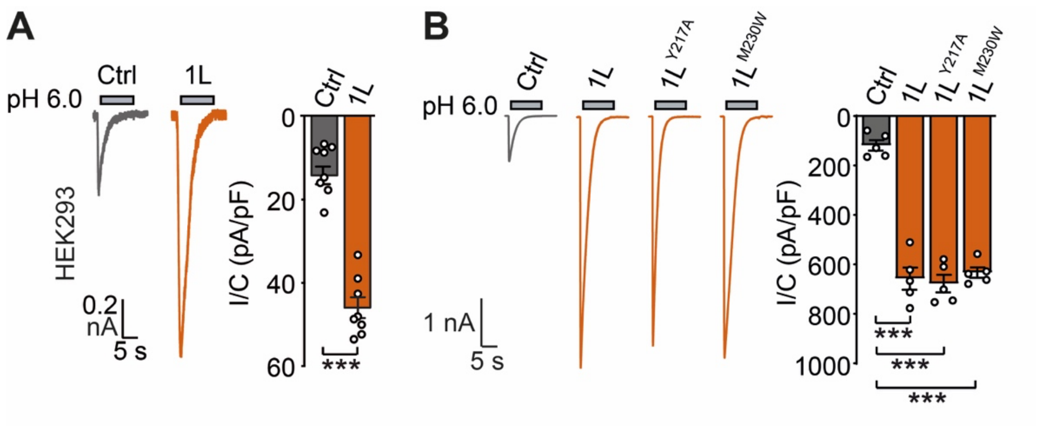

Fig. S3. PCYOX1L potentiates endogenous ASIC currents in HEK cells and the enzymatic function of PCYOX1L is not required for its effect on ASIC1a. (**A**) Representative traces (left) and quantification (right) of patch-clamp recordings of endogenous ASIC1a in HEK cells expressing GFP or PCYOX1L (n = 5). (**B**) Representative traces (left) and quantification (right) of ASIC1a co-expressed with RFP (n = 5), PCYOX1L (n = 5), PCYOX1L^{Y217A} (n = 5), or PCYOX1L^{M230W} (n = 6). Bars show the mean ± S.E.M. Data were analyzed by unpaired *t* test (A) or ANOVA (B). ***, $P < 0.001$.

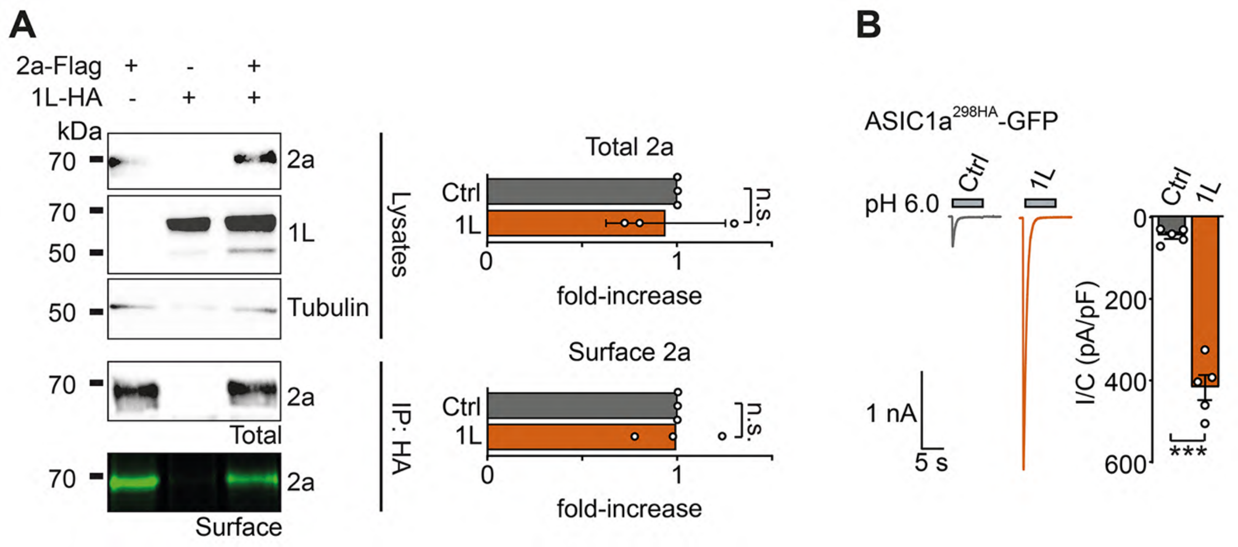

Fig. S4. Effect of PCYOX1L on ASIC1a/ASIC2a surface expression. (**A**) Representative western blot (left) and quantification (right) of ASIC2a-FLAG in the absence and presence of PCYOX1L-HA. ASIC2a was immunoprecipitated, separated by PAGE, and the total ASIC2a pool detected with an anti-FLAG antibody and the surface pool with an infrared dye (n = 3). Western blots at the top show protein expression. (**B**) Representative traces (left) and quantification (right) of ASIC1a^{298HA}-GFP co-expressed with RFP (n = 5) or PCYOX1L (n = 5). Bars show the mean ± S.E.M. Data were analyzed by unpaired *t* test. n.s., not significant, ***, $P < 0.001$.

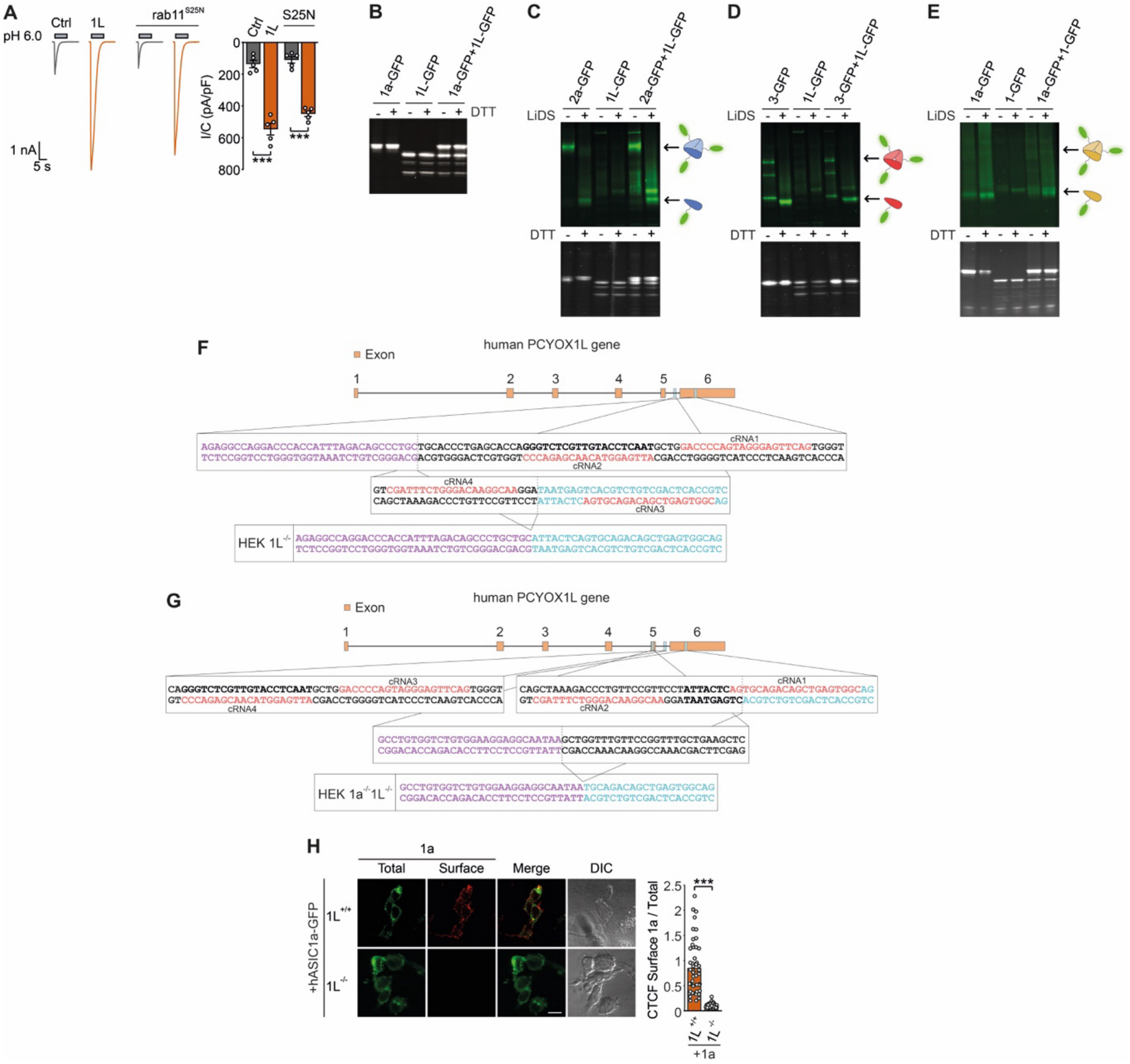

Fig. S5. PCYOX1L is not involved in slow recycling of ASIC1a and does not increase the abundance of ASIC2 or ASIC3 trimers. (**A**) Representative traces (left) and quantification (right) of patch-clamp recordings of cells co-expressing ASIC1a with RFP or PCOX1L; cells additionally expressed rab11^{S25N} as indicated (n = 5). (**B**) SDS-PAGE of samples used in Fig. 3E. Samples were treated with DTT as indicated. (**C to E**) High-resolution clear native–PAGE as in Fig. 3E, but with ASIC2a (C) or ASIC3 (D) instead of ASIC1a, or with PCYOX1 instead of PCYXO1L (E). ASIC trimers and monomers are indicated on the right. Lower panel, SDS-PAGE of the same samples as expression control. Samples were treated with DTT as indicated. (**F**) Genetic deletion of the *Pcyox1l* gene in WT HEK cells. Top, exon-intron structure of the human *Pcyxo1l* gene. Middle, position of cRNAs for CRISPR/Cas9 knock-out. Bottom, gene sequence after CRISPR/Cas9 knock-out. (**G**) As in (F), but for HEK *Asic1*$^{-/-}$ cells. (**H**) Confocal images of EGFP-ASIC1a-HA expressed in HEK293 *Asic1a*$^{-/-}$ (n = 47) and *Asic1a*$^{-/-}$/*Pcyox1l*$^{-/-}$ (n = 40) cells. Right, quantification of corrected total cell fluorescence (CTCF) of HA staining (surface ASIC1a) normalized to CTCF of EGFP staining (total ASIC1a). n = 2 independent experiments. Bars show the mean ± S.E.M. Data were analyzed by unpaired *t* test (A) and Mann-Whitney U-test (H). ***, $P < 0.001$.

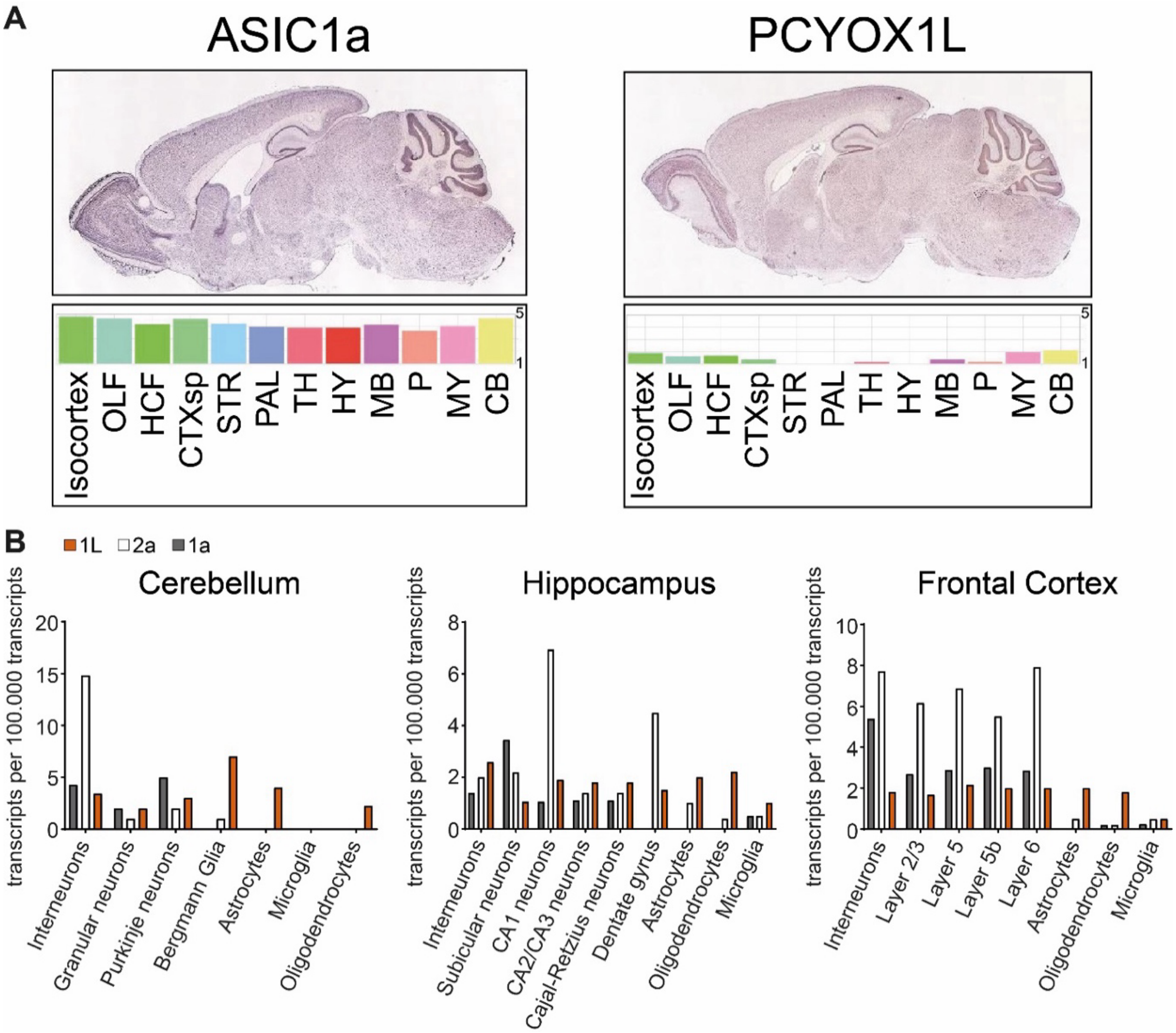

Fig. S6. PCYOX1L and ASIC1a are ubiquitously expressed in the brain. (**A**) Expression of ASIC1a and PCYOX1L in different brain regions according to the Allen Mouse Brain Atlas. Regions: Isocortex, Olfactory areas (OLF), Hippocampal formation (HPF), Cortical subplate (CTXsp), Striatum (STR), Pallidum (PAL), Thalamus (TH), Hypothalamus (HY), Midbrain (MB), Pons (P), Medulla oblongata (MY), Cerebellum (CB). (**B**) Expression of PCYOX1L (orange), ASIC1a (grey), and ASIC2a (white) in different cell types of the Cerebellum, Hippocampus and Frontal Cortex. Data were obtained from the single cell RNA sequencing database DropViz.

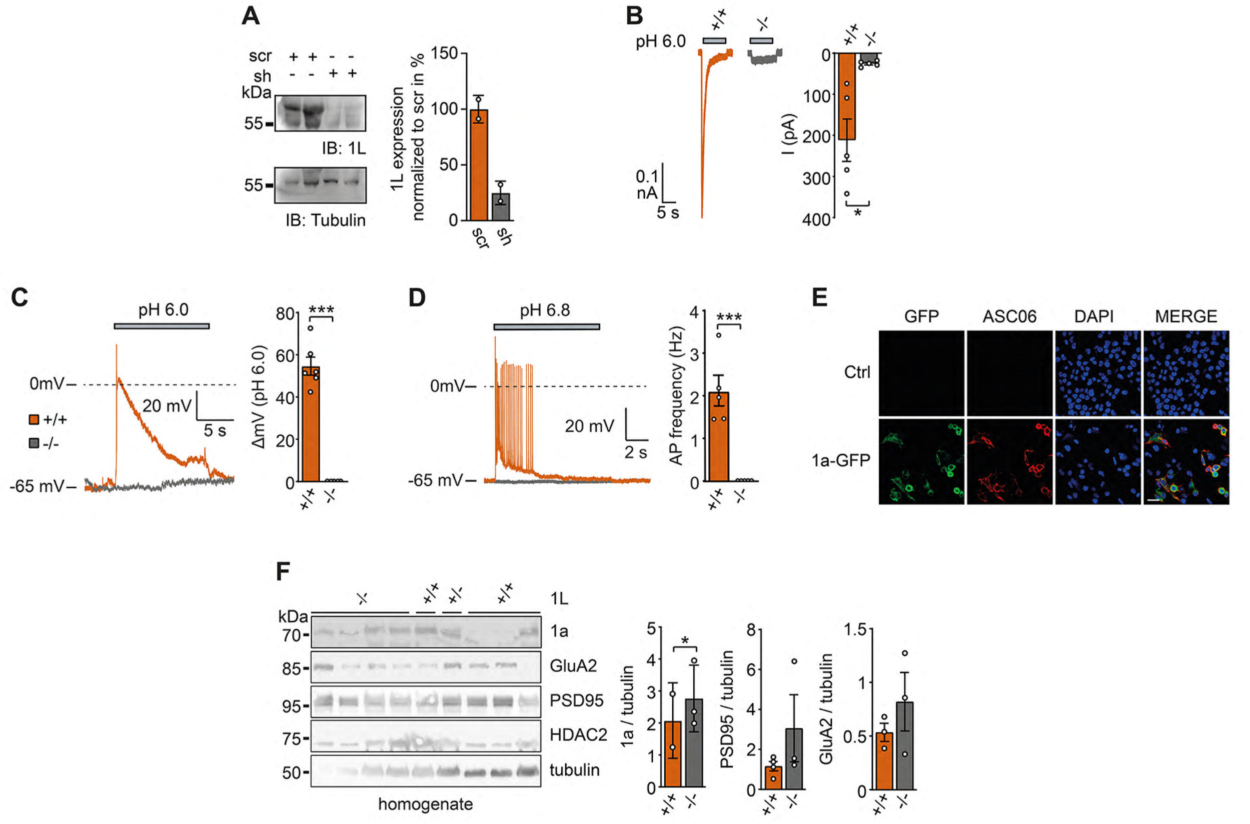

Fig. S7. Characterization of PCYOX1L in neurons. (**A**) Western blot of cerebellar granule cells (CGCs) transduced with AAVs carrying PCYOX1L shRNA (sh) or a scrambled sequence (scr) for 10 d. Samples were probed with an anti-PCYOX1L antibody (1L) and an anti-tubulin antibody (Tubulin) as loading control. Densitometric values for PCYOX1L were normalized to tubulin (n = 2 independent cultures from different WT animals). (**B**) Representative traces (left) and quantification (right) of patch-clamp recordings of cerebellar granule cells (CGCs) from WT or *Pcyox1l*$^{-/-}$ mice (n = 5). **(C)** Representative traces (left) and quantification (right) of current-clamp recordings in isolated cortical neurons from *Pcyox1l*$^{+/+}$ or *Pcyox1l*$^{-/-}$ mice (n = 6 for +/+ and n = 5 for −/−). Neurons were stimulated with pH 6.0. **(D)** as in (C) but stimulation was with pH 6.8 (n = 5). (**E**) Confocal images of nontransfected HEK cells or HEK cells expressing EGFP-hASIC1a, stained with ASC06 antibody (red). EGFP fluorescence in green, and DAPI in blue. Scale bar, 20 µm. (**F**) Western blot of homogenates as used in Fig. 4H. Right, protein abundance in homogenates relative to tubulin. Bars show the mean ± S.E.M. Data were analyzed by unpaired *t* test (A to D, and F). *$P < 0.05$; ***$P < 0.001$.

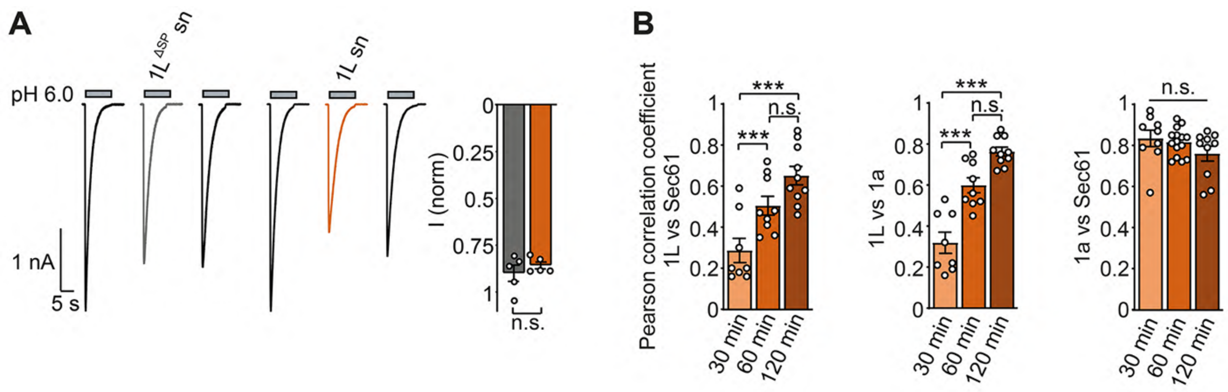

Fig. S8. PCYOX1L acts transcellularly. (**A**) Supernatants of cells expressing PCYOX1L or PCYOX1L$^{\Delta SP}$ were harvested and concentrated 10-fold. Cells expressing ASIC1a were patched and stimulated with pH 6. The same cell was incubated with the supernatant for 10 min and stimulated again. The amplitude after supernatant treatment was normalized to the mean of the preceding and the subsequent amplitude. Left representative current traces, right quantification (n = 5). (**B**) Cells co-expressing ASIC1a-GFP and Sec61-BFP were incubated with the supernatant from PCYOX1L-RFP expressing cells for 30, 60, or 120 min. Pearson correlation coefficients for the RFP, BFP, and GFP signals were calculated and compared (numbers of cells from left to right: 8, 9, 10). Representative images are shown in Fig. 4D. Bars show the mean ± SEM. Data were analyzed by unpaired *t* test (A) or ANOVA (B). n.s., not significant; *, $P < 0.05$; **, $P < 0.01$; ***, $P < 0.001$.
